# A novel application of X-ray computed tomography towards the characterization and interpretation of phase formations, mineral parageneses, and internal features in ancient copper slag from Tepe Hissar, Iran

**DOI:** 10.1371/journal.pone.0336603

**Published:** 2025-11-11

**Authors:** Benjamin Sabatini, Antoine Allanore

**Affiliations:** 1 Department of Materials Science and Engineering, Massachusetts Institute of Technology, Cambridge, Massachusetts, United States of America; 2 Center for Materials Research in Archaeology and Ethnology, Massachusetts Institute of Technology, Cambridge, Massachusetts, United States of America; Ataturk University: Ataturk Universitesi, TÜRKIYE

## Abstract

A selection of metallurgical slag artifacts from the Early Bronze Age site of Tepe Hissar, Iran, were characterized using X-ray computed tomography (XCT), X-ray fluorescence (XRF), X-ray diffraction (XRD), and optical and scanning electron microscopy with energy-dispersive spectrometry (SEM-EDS). The CT scans were used to identify regions of interest and internal features for sectioning, including pores, high-density inclusions, and differences in slag density and porosity distribution. The scans revealed internal features and patterns; however, contiguous metal-rich regions and thick surface minerals masked some features and misrepresented others. This study demonstrates how XCT enables the identification and collection of salient diagnostic information from slag artifacts before sectioning, providing a prescience of internal features and a volumetric 3D record of each artifact. After studying the 3D images, the artifacts were precisely sectioned and examined using optical microscopy, SEM-EDS, and XRD, revealing primary pyrometallurgical phases and secondary mineralizations, stratified slag layers, leaching channels, and internal microenvironments. Secondary precipitates and mineral parageneses within these environments are discussed, referencing the archaeological record, soil salinity, and Pourbaix diagrams. An explanation for the presence of speiss in some slag artifacts from Hissar is also detailed.

## Introduction

X-ray computed tomography, or, more colloquially, CT scanning, in archaeology and related fields, has dramatically increased in recent years since its first application to an Egyptian mummy in 1977 [1,2]. Since then, along with more nuanced internal scanning methods such as neutron diffraction [3–5](e.g., Cantini et al., 2024; Fedrigo et al., 2023; Festa et al., 2020), XCT has increased in frequency and application despite being logistically challenging to facilitate and cost-prohibitive [1,6–11]. In the last few years, newly imagined applications of CT, well beyond tangentially related medical purposes, have increased in number and expanded in scope to the study of ceramics [6], ancient glass [12], metal and metal statuary, mainly consisting of copper- and iron-based alloys [6,8,10,13–15], various human and animal remains, including but not limited to mummies [1,16,17], and prolifically, tree rings using high-resolution µ-CT to determine age and species [18–24].

The technique’s increased application and diverse use in the humanities and social sciences in recent years is undoubtedly due to its ability to impart internal structural information non-destructively [1,6,11,14,17]. This aspect of XCT is well-suited for studying irreplaceable cultural objects, spurring the development of cost-reducing, custom-made XCT apparatuses, software, and portable scanners designed for on-site analyses. A handful of such devices have been developed to overcome size constraints, for instance, when scanning a large, dense cast-iron engine block, or, conversely, nanoscale samples of wood [8,24], all while offsetting the costs of medical-grade CT scanners and the impracticality of transporting invaluable and delicate artifacts to medical facilities [7,9,10,13,25]. These recent trends indicate a growing desire to utilize CT across various fields, and with the advent of industrial scanners, it has become possible to produce higher-quality scans at lower cost with greater versatility and adaptability.

Contrary to its hallmark, this study employs XCT as a preliminary step in the destructive analysis of slag artifacts. Three-dimensional volumetric images may provide invaluable information on internal diagnostic pyrotechnological features, such as charcoal, prills, and porosity, as well as their relationship to external phenomena, including corrosion and cracks. This method may also enable the documentation of artifacts and the precise targeting of features for sectioning. Without these data, metallurgical slags are typically cut, and portions are pulverized after surface observations and chemical analyses have been performed with optical microscopy and X-ray Fluorescence (XRF) [26–28], respectively. Following preliminary characterization, sets or typologies and representative specimens are selected based on inferential sectioning processes, where superficially observed and sometimes chemically analyzed features, such as color, texture, geometry, and corrosion patches, serve as indicators of internal structures and chemical phenomena. After sectioning, fortuitously discovered pores, inclusions, minerals, and metal phases are then documented and studied [29–32].

Archaeological slag, however, is a highly inhomogeneous and altered product, containing glassy, crystalline, and fused phases, unreacted ore minerals, varied textures [26,29,32–37], and a host of secondarily precipitated minerals and compounds [34,38–42]; their mineralogical and pyrometallurgical descriptions are, therefore, incredibly challenging. And while the inferential technique described above has been adequate, as this study will show, the lack of prescience regarding the specimens’ internal structure inherently means that even well-informed inferential sectioning will miss diagnostic features such as metal droplets, overlaid surfaces, and trapped inclusions. The implications are that processual and procedural information may be unaccounted for, or multiple sections might be needed to achieve the same level of knowledge imparted by structurally detailed 3D volumetric images. In short, destructive analyses of slags that rely on traditional investigative techniques may not capture the most salient aspects of the pyrotechnological processes and conditions that produced them. Indeed, the surface of slags, having been exposed to various environmental conditions over several centuries or even millennia, alters, resulting in external crusts and coatings that may be unreliable indicators of internal structures and compound and mineral paragenesis [34,39].

The slags analyzed in this study originate from Tepe Hissar, Iran, and had been studied, as part of a larger corpus, by Thorton (2009), who determined their date by radiocarbon to periods IIB–IIIB (c. 3350–3000 BCE) and the late Chalcolithic to Early Bronze Age (Table 1) [43–47]. These dates were further refined based on pottery typologies to ca. 3100–2900 BCE [48]. Several of the slags were found to contain small speiss prills [43,49], which, by modern-day definition, are an undesirable byproduct; however, archaeologically, they are a significant outcome [50–53]. Understanding the processual circumstances leading to their formation, presence, and origins was the impetus for revisiting the slag artifacts; however, the focus of this article is primarily on the application of XCT. Nevertheless, speiss is discussed in a limited capacity because it has been a point of contention in the field, particularly concerning these specific artifacts. Speiss in archaeometallurgical contexts is defined as a di-iron arsenide (Fe_2_As), iron arsenide (FeAs), or iron di-arsenide (FeAs_2_) that lacks base metal and is theorized to have been intentionally produced, traded, and used as an additive to make arsenical copper as early as the 4^th^ millennium BCE [44,47,49,52,54–58]. Understanding its formation in ancient smelting contexts is therefore of the utmost importance for deciphering prehistoric metallurgical practices; however, this question will only be briefly addressed in this work [59–61].

**Table 1:** Findspot, time period, and identifying details for the studied slags.

Museum no.	Field no.	Findspot	Plot	Area	Lot	Stratum	Mass (g)	Stage	Time Period	Date (BCE)
76-30-83	H76-S37A	NE	CG90	P	12A	> 5	45.8	D	Mid – IIB	3100–3000
76-30-91	H76-S45B	NE	CG90	P	12	2	63.5	D1 – C2	IIB – IIIB	3100–3000
76-30-35	H76-S39	NE	CG90	5	13	7	342.5	C2	Early – IIIB	3100

The dates for each were determined by Thornton (2009) and Thornton et al. (2009) using radiocarbon dating and later refined by Gürsan-Salzmann (2016) based on pottery assemblages to approximately 3100–2900 BCE. The listed dates reflect the later refinement within the range given by Thornton.

A small selection of slags from Tepe Hissar, Iran, dating to the 4th millennium BCE, was loaned to Professor Allanore at MIT in 2022 by the University of Pennsylvania’s Penn Museum [LOAN AGREEMENT LO-2022–7 ST-2022-14], located in Philadelphia, PA. The Museum later granted permission to section three of the artifacts in 2023, corresponding to two types: H76-S37A and H76-S45B, which are assuredly rich in copper and covered in green corrosion, and H76-S39, which potentially has no copper but may contain speiss [43,44,49,62]. No permits were required for the described study, which complied with all relevant regulations. They originate from a collection of metallurgical materials excavated in 1976 with support from the Iranian Center for Archaeological Research in Tehran by the Penn Museum and the University of Turin under the direction of Robert H. Dyson and Maurizio Tosi [63,64]. From these materials, slags H76-S37A and H76-S45B also have embedded charcoal and large exposed voids and pores. Comparatively, slag H76-S39, a possible tap slag with a flowed and layered appearance, has a smooth upper surface and a rough underside with far smaller pores [27,30,65]. These slags were selected for XCT and sectioning. Their findspot details and dates are given in Table 1.

## Materials and methods

Before CT scanning, following typical study practices, the slags were described by texture, color, shape, mass, size, and relative magnetism [29,30,32]. After these basic characterizations, their surfaces were chemically analyzed using X-ray fluorescence (XRF), followed by XCT to identify internal features and determine their relationship to external phenomena, such as pores and corrosion products. The scans showed clear density differences and internal patterning, which guided later sectioning. Following sectioning, the exposed surfaces of the slags were ground and polished, followed by the imaging and chemical analysis of select features using optical, X-ray diffraction (XRD), and scanning electron microscopy with energy-dispersive X-ray spectrometry (SEM-EDS). In brief, XCT was integrated into a metallurgical slag characterization and sampling workflow, enabling precise selection and sectioning of internal features. Specific features identified by XCT were studied in greater detail after the specimen was sectioned.

### Surface elemental analysis

A Thermo Scientific Niton XL3t GOLDD+ portable XRF (pXRF) was used to acquire surface chemistry data. The device was set to “AllGeo” mode, and the data were exported using the provided NDT software. The acquired elemental data were later converted to oxides typically found in copper smelting slags [32,40,66]. The artifacts were analyzed on exposed flat surfaces, rather than over crevices or angular areas, avoiding obstructions where the analyzer’s objective window could sit flush. The XRF data compiled in this work are given in Table 2. They show noticeable variation between analyses, reflecting the typical compositional inhomogeneity of ancient slag and slag surfaces [30,65,67], and to some degree, the absence of several elements. Of note, slag H76-S39 had been analyzed previously using a Spectrolab 2000 ED-XRF and found to contain Na_2_O, TiO_2_, SrO, and BaO at 1.22, 0.33, 0.11, and 0.15, respectively [43], which were not detected in the current XRF analyses. This data is also shown in Table 2.

**Table 2:** X-ray fluorescence analyses of slags H76-S39, H76-S37A, and H76-S45B converted to oxides and given in wt. %.

*Artifact No.*	As_2_O_3_	CuO	FeO	CaO	Al_2_O_3_	P_2_O_5_	SiO_2_	Cl	SO_3_	FeO:SiO_2_
H76-S39^*^	0.43	0.01	54.81	4.8	5.56	0.31	28.74	0.11	1.85	1.91
H76-S39	3.56	0.77	56.99	3.50	3.46	0.19	16.7	0.72	10.37	3.41
H76-S39-2	0.27	0	52.03	4.13	7.64	0.30	33.33	0.10	1.38	1.56
H76-S39-3	6.13	0.04	58.25	8.75	1.23	9.92	9.16	0.19	1.10	6.36
H76-S37A	0.03	5.47	25.92	10.75	8.70	0.32	41.02	0.73	2.02	0.63
H76-S45B	0.12	43.67	11.68	6.14	3.64	0.20	25.12	5.72	2.34	0.47
H76-S45B-2	0.62	63.73	2.73	4.02	1.97	0	6.32	8.69	11.44	0.43

Postfixed numbers indicate additional analyses of the same slag, and ‘*’ a prior analysis by Thornton (2009) (*see* S2 Appendix for the complete set of analyses).

### X-ray computed tomography

Each slag was packed into secure craft foam holders and imaged using a Lumafield Neptune CT scanner. Slags H76-S37A and -S45B were scanned at 120 kV for 151 min using a 1 mm Cu filter, having less dense, non-glassy outward appearances, with an exposure time of 8.2 s; H76-S39, being far larger with fewer pores, thus being more dense and possibly glassy, was scanned at 190 kV for 339 min with a 6 mm Cu filter and exposure time of 20.2 s. The acquired data consists of a series of two-dimensional (2D) radiograph projections arranged in sequence as the artifacts were rotated on top of the stage within the sample chamber [1,11,68]. The radiographs are then synthesized to create 3D representations, incorporating the relative density data of the scanned object in voxels, or 3D pixels. Since each voxel has an attenuation value, expressed in Hounsfield units (HU) within the total volume, they can be selectively isolated based on these values and viewed separately in Voyager, the accompanying browser-based software [69–71]. The contrasting voxels created a 3D internal map of the slags, showing the precise locations of internal features. These locations guided the sectioning of the slags.

Denser materials, such as metals, strongly attenuate X-rays and consequently exhibit higher HU values [4,14], while less dense materials, like voids or organic matter, are lower [3,10,72,73]. Based on their attenuation, several features of interest were chosen for sectioning, including those expected to be metal droplets, which show the highest relative density in the slags, and less dense regions thought to be charcoal, technical ceramics, or other low-density materials. Since CT scans only display differences in density, suppositions of the internal features were made based on prior studies of slag from the region [26,28,44,54,56,59,60,74,75], protruding features such as charcoal, and the 3D-rendered shapes and distribution of these features. The slags were sectioned with a diamond-tipped blade and water lubricant along precise X, Y, and Z coordinates. After sectioning, the surfaces were polished using kerosene down to 1 µm using diamond paste in preparation for imaging and chemical analysis.

### Optical and EDS characterization

Optical images of the exposed features were captured in both light- and dark-field using a Leica DM/LM reflected light microscope (type 020–520.008), an attached Axiocam 305 color camera (5 megapixels), and ZEN Core 3.6 software at magnifications ranging from 50 to 500 µm. Following optical microscopy, additional images were collected, and chemical analyses were performed using a JEOL JSM-6610LV SEM operated at 10–20 keV, equipped with a Silicon Drift EDS detector. Chemical quantifications were performed using Iridium Ultra software.

### Mineral and phase identification

After chemical analysis, the powder from each precision-cut sample was filtered and dried for XRD analysis using a PANalytical Empyrean diffractometer equipped with a GaliPIX3D detector. The device was equipped with a molybdenum MoKα radiation source (λ = 0.7107 Å) operating at 60 kV and 40 mA, scanning from 5 to 60° 2θ with a step size of 0.014 ° 2θ at a rate of 150 s/step for 22 minutes. Mineral and phase identification were performed using HighScore Plus (v. 5.1.0) with the ICDD PDF5 + Converted database, utilizing Rietveld refinement based on the major phases identified in each slag, given the proportion of amorphous phase in each powder sample. Refinement can significantly alter XRD spectra, potentially misrepresenting phases and minerals, necessitating corroborating chemical and structural analyses.

### Phase and mineral alteration Eh-pH (Pourbaix) diagrams

The stability of pyrometallurgical phases and relic minerals in the slags, considering regional and localized environmental and macroscopic conditions, was thermodynamically calculated and presented in Eh-pH (Pourbaix) diagrams. The influence of soil salinity on secondary precipitate formation, based on the phases and minerals found in the slags, is highlighted in respective diagrams, as it dramatically affects paragenesis. Soil conductivity surveys conducted at Dāmghān in the Semnan province, Iran [76,77], provided an estimate for chloride activity (*γ*) and the soil solution’s ionic strength (*IS*), accounting for non-ideal behavior at higher concentrations. The salinity, measured at 4830 μS/cm, was converted to Total Dissolved Solids (TDS) by multiplying it by an empirical conversion factor of 0.65. Assuming the majority of dissolved solids were NaCl, *IS* is equal to its molar concentration (*c*_*i*_) at ~ 0.054 mol/L, according to Equation 1. The extended Debye-Hückel equation was then used to determine *γ*, with parameters *A* ~ 0.51 and *B* ~ 0.33, ion size parameter *a*_*0*_ = 3.5 *Å*, the net charge of Cl^-^ (*z*_*i*_), and *IS* at 273.15 K and 1.4 bar, approximating the pressure at 3 m below the surface (Equation 2). The Cl^-^ activity was then calculated using *IS* and the logarithm (base 10) of *γ*, resulting in a value of −1.3626, which was used whenever chlorine-containing species, phases, and minerals were present.


$$IS=\frac{\text{1}}{\text{2}}\sum c_{i}z_{i}^{\text{2}}$$ (1)

$${log}_{\text{1}\text{0}}\gamma =\frac{-A|z_{+}z_{-}|\sqrt{IS}}{\text{1}+Ba_{\text{0}}\sqrt{I}S}$$ (2)

Furthermore, for each Pourbaix diagram, several adjustments were made to the activity of contributing species to account for the in situ conditions of the slags, given their geographical location just south of the Zagros Mountains and proximity to the Caspian Sea [77–79]. The activity of carbonates in soil air was set to −3 to meet the minimum partial pressure required for the formation of siderite [80,81], as identified by chemical and XRD analysis. In some instances, the presence of minerals and phases dictated adjustments to the activities of species; however, unity was assumed for arsenic and copper, given their ubiquity in some slags and the presence of copper-based sulfides in others, and unity activity for H_2_O.

The predominance diagrams were calculated with published datasets containing Gibbs energy of formation ${(\Delta G}_{f}^{\text{0}})$ parameters for the discovered minerals and compounds. These data were used to calculate the standard molal thermodynamic properties and chemical affinities of reactions using the open-source CHNOSZ R package to create Eh-pH diagrams [82]. The data used for these calculations [83–85], which consist of calculated parameters based on the revised Helgeson-Kirkham-Flowers (HKF) equation for aqueous species, were selected for their suitability to the slag burial environment and the rigor of their data evaluation. Several supplementary data sources were also added to account for As-containing minerals and aqueous species, and updated thermodynamic parameters [58,86–97]. The scripts and data used to produce the diagrams are available in the S3 and S4 Appendices.

## Results

### Macroscopic and archaeological interpretation

Visual inspection of the ‘green slags’ H76-37A and S45B, in addition to the subsequent XRF analyses, correlated with prior scholarship [56,98,99], affirming they are products of copper smelting, showing substantial green corrosion and charcoal among highly porous, grey-textured surfaces [29,32]. Slag H76-S39, however, is noticeably darker, with reddish-brown corrosion along its top surface; nevertheless, it is believed to have derived from the same high-temperature smelting process. Although not studied in this current work, slag H76-S30, a tap slag containing small amounts of iron (di)arsenide speiss (FeAs and FeAs_2_), is chemically similar and stated to have come from the same smelting operation [43]. Slag H76-S39 was therefore labeled a ‘speiss slag’ thought to have been adjoined to H76-S30, but, having cooled slowly, conditions did not prevail to form speiss [43,49].

**Fig 1:**
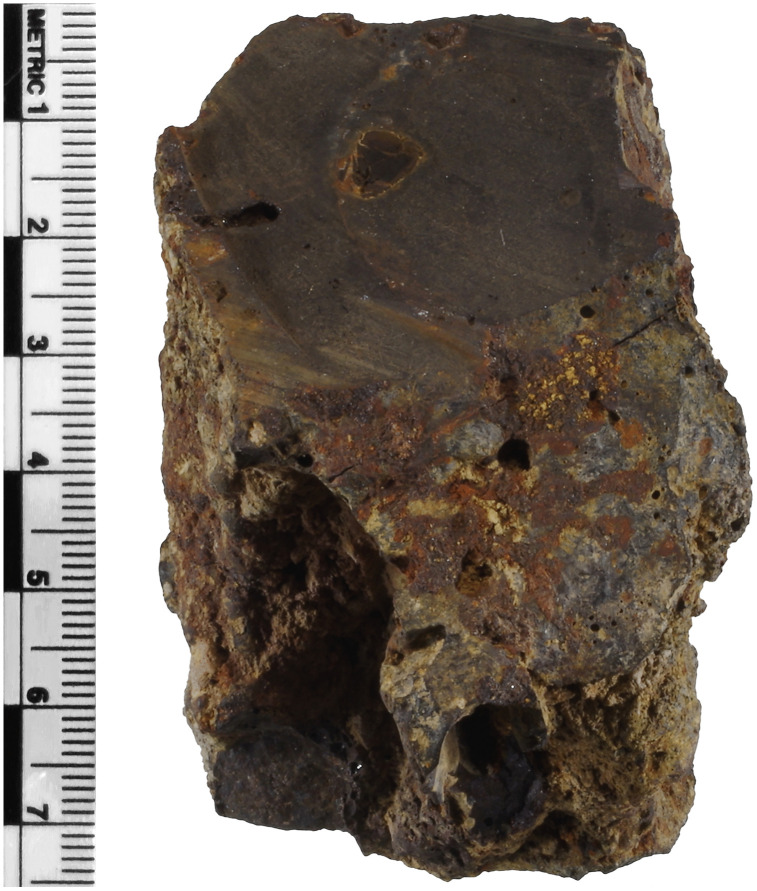
Previously sectioned surface of slag H76-S39 showing an entrapped charcoal inclusion embedded within a second layer. Based on the outer texture of the slag, the left side represents the bottom, and the opposing side represents the weathered or altered top. X-ray fluorescence analyses revealed that the top layer exhibits increased concentrations of arsenic, likely resulting from the release of arsenic from the slag matrix and pyrometallurgical phases during alteration. Additional images are provided in S1 Appendix.

Slag H76-S39 is dark in color and weakly magnetic, with a smooth top surface, a flow-shaped midsection, a discernible crease along the midsection, and a rough underside, where it likely settled on soil. Iron corrosion products cover portions of the top surface, along with more extensive localized surface disruptions. Notably, this slag had been previously studied and sectioned; its bulk, having been exposed, appears very dense, with most pores being less than 1 mm in diameter, a few ranging from 1 to 5 mm, and a ~ 1 cm unreacted charcoal inclusion (Fig 1).

**Fig 2:**
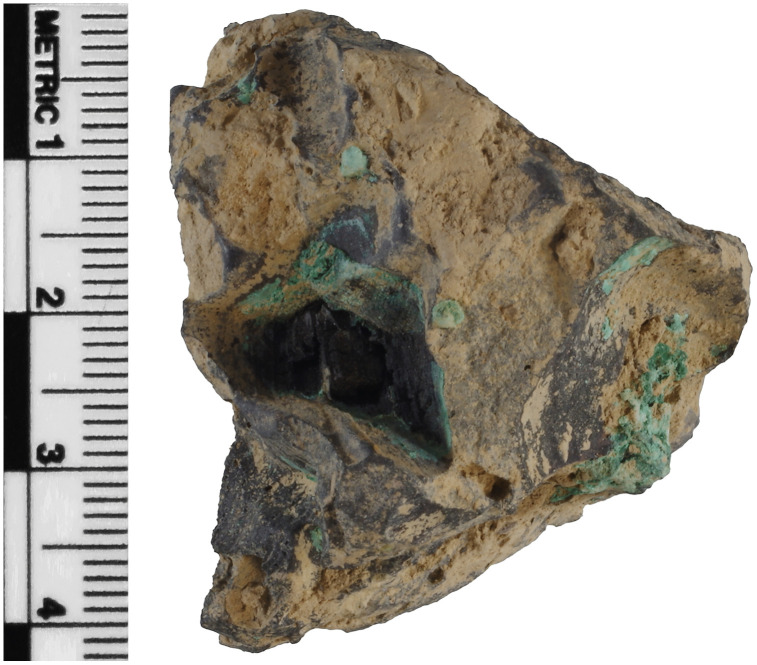
Slag H76-37A showing a triangular prism shape with a triangular charcoal inclusion, green corrosion, and surface-deposited clay from the burial environment. Additional images are provided in S1 Appendix.

**Fig 3:**
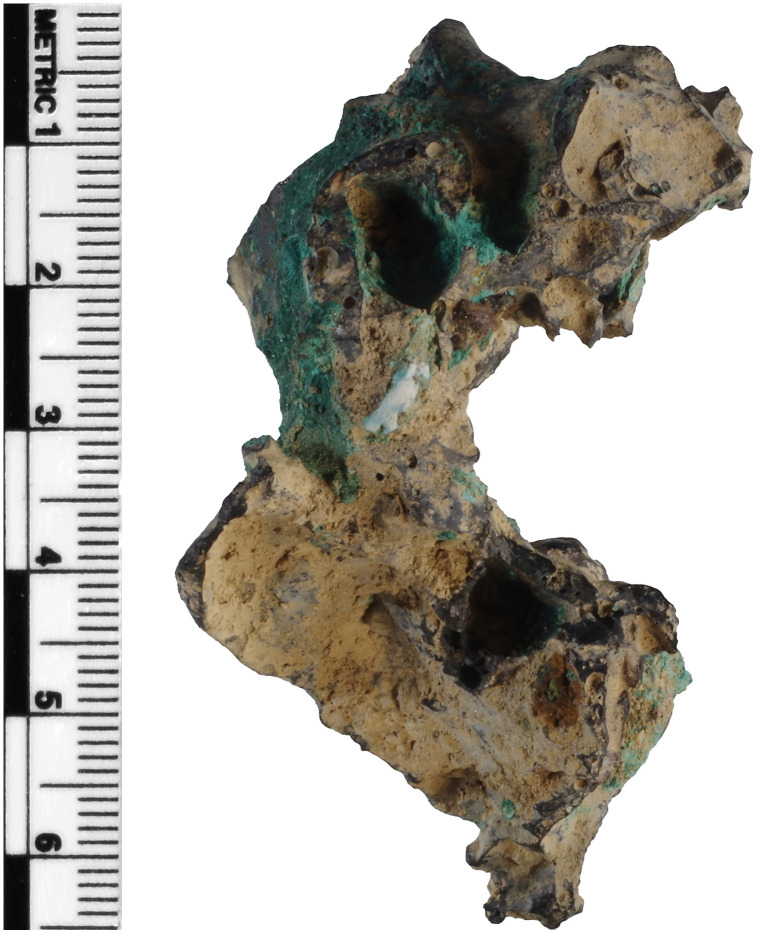
Slag H76-S45B, with its distinctive curved shape, exhibits possible embedded pyrotechnic ceramic at the bottom, large pores, charcoal inclusions, and copious green corrosion and surface-deposited clay from the burial environment. This slag is theorized to have been wrapped around a cylindrical object, possibly a tuyère. Additional images are provided in S1 Appendix.

Slags H76-37A and H76-45B can be broadly attributed to copper smelting. Copious copper oxide and chloride corrosion cover much of their surfaces where sediments have not adhered, suggesting appreciable amounts of copper metal or phases. Slag H76-S37A is moderately magnetic and triangular prism-shaped with large inclusions, such as charcoal, and significantly large and angularly or teardrop-shaped pores on every side that, in some instances, extend beyond ~ 1 cm at their widest (Fig 2). Its bulk is grey and far less dense than slag S39, considering the high proportion of pores, likely having formed in a different part of a smelting apparatus [29,100–103]. This slag and H76-S45B have been weathered extensively through various pores and cracks. They also contain unreacted inclusions of charcoal, gangue, quartz, ores, and associated minerals [29,36,102]. Slag H76-S45B is weakly magnetic but appears denser despite having many relatively large pores. It also has a smooth-flowing red surface, likely cuprite (Cu_2_O), and is covered in clay and sediment. Interestingly, the slag contains embedded pieces that appear to be from a furnace lining or other pyrotechnic ceramic (Fig 3). Its shape also suggests it may have been wrapped around a cylindrical or round object, possibly a tuyѐre or stone.

These slags have all been associated with related copper smelting operations, despite being found in disturbed levels of the site [62,104]. Their outward and inward appearances, in the case of H76-S39, inform about their role and how they were coerced into existence from compositionally complex copper ore smelting operations conducted in the early 4^th^ millennium, Iran [29,46,59,74,105–108].

### X-ray fluorescence surface analyses

Slag H76-S39 showed no visible copper or copper corrosion and is likely a tap or hearth slag, suggesting that high temperatures and low viscosity were achieved during smelting [59,66,109–111]. The other slags, H76-S37A and H76-S45 B, are likely the result of incomplete copper/slag separation, as evidenced by their large pores and significant copper patina among unreacted inclusions [30,105,112]. X-ray fluorescence analyses confirmed high concentrations of Cu, Al, Si, S, Ca, and Fe oxides in both, agreeing, in complexity, with prior studies of slags from the site [43,49]. The Cl concentration on the surface of H76-S45B is of particular importance, as it is likely due to high-salinity weathering and the deposition of secondary corrosion products [30,32]. For instance, secondary surface enrichment of atacamite or paratacamite (Cu_2_Cl(OH)_3_) can occur due to coprecipitation and adsorption near saltwater bodies, whereby the precipitation of chlorides sequesters released copper [32,40]. Ordinarily, some Cl is present in copper smelting slag, especially if polymetallic ores, atacamite, and gangue minerals had been smelted [30,100,113], but its concentration would be mere fractions of a percent compared to those shown in the surface analyses. Analysis of slag H76-S37A revealed significantly less copper than iron oxide, at 5.47 and 25.92 wt.%, respectively. High Fe concentrations are not without precedent for slags at the site [43,49], and are likely due to higher concentrations of fayalite (Fe_2_SiO_4_), magnetite, and hematite (Fe_2_O_3_) at the analysis location [66,114–118], and an overabundance of Fe in the Cu ores [26,66,67].

The largest slag in the set, H76-S39, also showed high CaO, and, along the top, in the region visually identified as iron corrosion, were significant concentrations of arsenic trioxide (As_2_O_3_) at up to 6.13 wt.%. While this percentage of As is not within the range of an iron arsenide speiss, it suggests that substantial amounts of As, and perhaps speiss, resided just below the surface, or that the slag was in contact with an external source of As. Given the disrupted appearance of this region, however, the high As concentrations are likely due to weathering, leaching, and reprecipitation [39,119,120]. The high concentration of CaO in the corresponding analysis may have also contributed to the increase in As, as it has been shown that higher slag alkalinities promote the formation of stable calcium arsenates, such as Ca_3_(AsO_4_)_2_, increasing As content by as much as 20–50% in modern-day copper smelting operations. Similarly, an increased Fe[O]:SiO_2_ ratio, which is the highest in this analysis, can lead to a reaction between Fe_3_O_4_, or Fe_2_O_3_, under oxidizing conditions with As_2_O_5_ to form ferric arsenate (FeAsO_4_) [121,122]. These reactions and others like them are responsible for increasing the concentration of As in the slags.

### X-ray computed tomography

**Fig 4:**
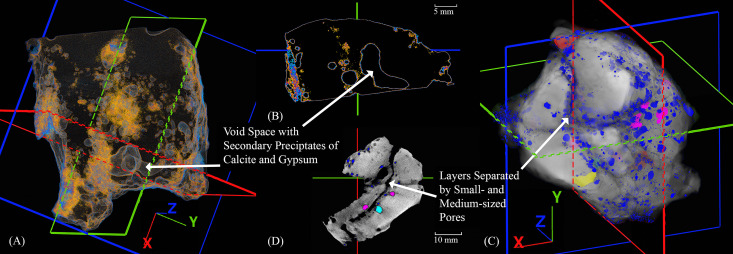
Three-dimensional and 2D XCT images of slag H76-S39. The crosshairs shown in RGB correspond to X, Y, and Z coordinates and intersect precisely with features selected for further analysis and characterization. (A) Transparency of the slag delineating internal pore distribution, including one large, irregularly shaped void that was sectioned along the X-axis. (B) A 2D image perpendicular to the X-axis, where the slag was later sectioned. (C) A three-dimensional image with slight transparency shows the location of small and medium-sized pores that formed between successively deposited layers of slag. (D) A two-dimensional image perpendicular to the Z-axis showing density differences and spaces between layers, associated porosity, and where the layers and cracks intersect.

X-ray Computed tomography confirmed the internal porosity first observed along the previously sectioned surface of slag H76-S39 (Fig 4). Furthermore, additional internal features and patterns were revealed, showing the precise location of diagnostic features despite the slag’s high-density matrix and brown exterior corrosion, which attenuate X-rays. Given that this is a tap slag, the scan showed regions of high porosity along its top and bottom, which appeared as small vacuoles or pores where the slag flowed onto the soil, stone, or perhaps other slag or technical ceramic. These pores can form when the molten slag meets a cooler material that contains moisture [30], which rapidly vaporizes and cools the slag, causing localized porosity [34,40,66,123]. Far smaller pores also appear on the surface of the slag, varying from 0.00241 to 0.289 mm³; however, these are likely the result of weathering [32,40].

Slightly larger median-sized pores, between 0.289 and 0.574 mm³, are also located on the surface and form a semi-contiguous band across the midsection of the slag. Larger still, pores greater than 0.569 mm³ appear internally near the midsection. The clustering of median-sized pores along the midsection and uneven stratification are discernible (Fig 4C and 4D), suggesting that the slag formed from two layers, where a first, cooled and hardened layer could have met a consecutive one or folded onto itself in a continuous molten pour. Prior visual observations support the likelihood that this sequence of events occurred. In addition, the exposed charcoal inclusion and the congregation of tiny pores around it, further support the supposition that the slag settled in layers. The charcoal sits encased in the second, or top layer; once encased, as the slag cooled, decreasing temperatures would have prevented it from reacting further [110,114,118,124,125]. The largest feature identified by CT was, at its widest, 3.49 mm (Fig 4A and 4B). It appeared slightly denser than the pores, hinting that it contained X-ray attenuating materials. Slag H76-S39 was sectioned along the coordinates of this feature, which also bisected the external As-rich surface identified by XRF analysis.

**Fig 5:**
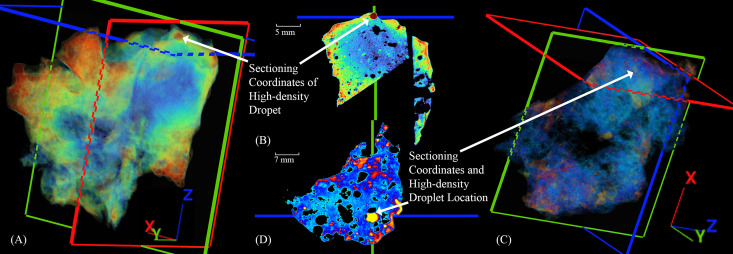
Two-dimensional and 3D images of slags -S37A [(A), (B)] and H76-S45B [(C), (D)], respectively, showing the slice planes in software before sectioning. (A) A high-density metal droplet from slag H76-S37A, ~ 1 mm in diameter and chosen for sectioning, is shown in 3D near the exterior of the slag. (B) The image and coordinates of a high-density droplet pre-section are shown perpendicular to the X-axis. (C) Location of a high-density droplet in slag H76-S45B and the overall 3D image. The droplet resides near the exterior of the slag, where it settled above a location presumed to be a cylindrical object or tuyère. (D) Image of the pre-sectioned location of a high-density droplet perpendicular to the X-axis.

**Fig 6:**
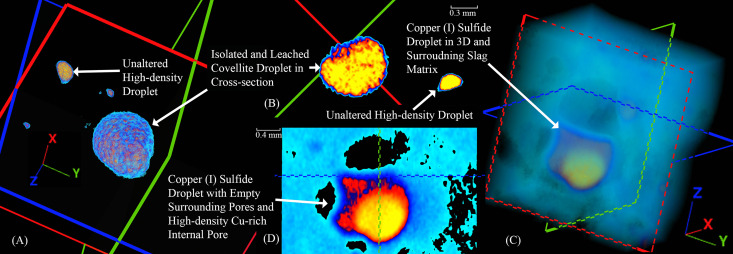
Two-dimensional and 3D images of slags -S37A [(A), (B)] and H76-S45B [(C), (D)], respectively, showing internal metal droplets. (A, B) Cross-section of the large covellite droplet showing variably dense but distinguishable phases. In contrast, note that the smaller, unaltered droplet immediately next to it, which has no connecting conduits, is contiguous and shows no signs of leaching or corrosion. (C, D) CT scan aligning with the sectioned droplet. Note the three external pores and the high-density internal pore of the droplet. The internal pore has a surface layer of cuprite, which attenuates the X-rays from the CT scan, giving it a more intense red color.

The CT scans of slags H76-37A and H76-45B showed a striking difference in their internal pore distributions compared to H76-S39, in addition to several high-density internal features and surface corrosion products (Figs 5 and 6). Slag H76-S37A exhibited surface corrosion with a similar relative density to the presumed internal copper-containing droplets that had congregated together (Figs 4A–4D). Since the surface corrosion had previously been identified as atacamite/paratacamite, the internal droplets were thus likely to contain chlorides or other impurities. One of the largest of these high-density droplets, a nearly perfect sphere ~ 1.5 mm in diameter, was later targeted for sectioning. Based on the distribution of the larger droplets, although size and shape are not necessarily determinative of composition or process [102,109], its location suggests that it resides near the bottom of the slag. Due to gravity, larger copper droplets tend to settle, and while viscosity, surface tension, and unreacted inclusions may impede their descent [109,126], this droplet, along with similarly dense droplets nearby, appeared to have migrated downward. Slag H76-S45B contains similarly spherical droplets, in addition to ovoid and elongated ones, which appear as semi-circular cutouts in 3D. However, in this instance, they are possibly composed of copper, copper sulfides, or matte, based on the varied amounts of S and Fe shown by the XRF analyses (Fig 5A and 5B. The corroded surfaces and internal features are similarly dense and thus challenging to differentiate in the CT scan; however, the droplets are slightly more attenuated. Assuming the internal high-density features are copper droplets, high-density sulfides, or matte, their size and distribution suggest the green corrosion-rich surface is the top of the slag. The droplets would have settled, being denser [127], on the upward-facing inner border of the semicircle, which was theorized to have been formed by a tuyѐre. However, unlike H76-S37A, this slag has randomly distributed pores of various sizes, ranging from 0.00249 to 31.1 mm³, suggesting that the voids were produced under conditions that were efficient at evolving gases, such as SO_2_, rather than from contact with a lower-temperature material. Since this slag contains a multitude of similar high-density droplets, only one was targeted for sectioning.

### Microscopy and X-ray diffraction

**Fig 7:**
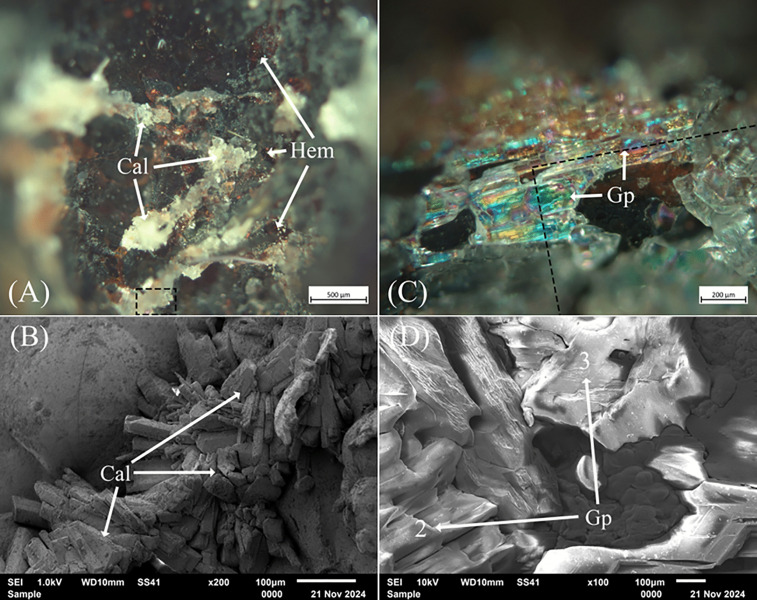
Optical images of calcite (Cal) and gypsum (Gp) secondary precipitates were found within the large, bisected pore of slag H76-S39. (A) Opaque and nested calcite with interspersed hematite (Hem) crystals. The black outlined boxes in (A) and (C) correspond to the SEM image and analysis location in (B) and (D), respectively. (B) Secondary electron image and broad EDS analysis corroborating calcite mineralizations. (C) Prismatic and multi-hued gypsum crystals. (D) Secondary electron image and broad EDS analysis confirming gypsum. Of note is that calcite or gypsum precipitated in the pore but never in the same location. Mineral abbreviations are from Warr (2021) [132].

**Fig 8:**
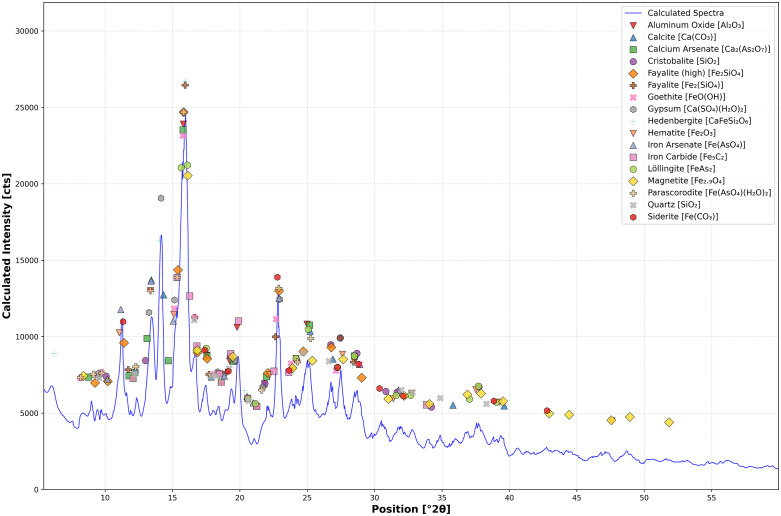
X-ray diffraction spectra of slag H76-S39. Several detected phases and minerals, identified via optical microscopy and EDS, were used for Rietveld refinement of the spectra. Aluminum oxide, calcium arsenate, cristobalite, iron arsenate, iron carbide, löllingite, and siderite were not seen in significant quantities by analysis, suggesting they may exist but in limited proportions throughout the slag. Due to the large percentage of amorphous phase in the slag, many mineral-defining peaks may have also been obfuscated.

The largest pore in H76-S39 was initially presumed to be charcoal or technical ceramic, having a slightly higher density than the externally exposed inclusion; however, after sectioning, it was revealed to be a void lined with dense iron-rich magnetite, hematite, or oxyhydroxide phases and minerals (Table 3), within which were opaque and prismatic crystals. These secondary calcite and gypsum mineralizations, although prominent in the optical images, were not visible in the CT scans due to their small size and attenuation caused by the iron oxide-rich lining [128,129]. Optical microscopy revealed opaque rhombohedral and colorless crystals, with the latter showing multi-colored hues [130,131], suggesting calcite (CaCO_3_) and gypsum (CaSO_4_·2H_2_O), respectively (Fig 7A and 7C. Energy-dispersive spectroscopy corroborated calcite and gypsum stoichiometrically, along with minor concentrations of Na, Al, Si, P, K, and Sc, which, in combination, could have instilled variegated blue, green, orange, yellow, and purple hues (Table 3). The detection of Fe was associated with interspersed hematite and mixed weather-derived secondary precipitates, with the former appearing ruby-red under optical microscopy (Fig 7B and 7D). These minerals were also confirmed by targeted EDS, which revealed Si, Ca, and Cu impurities, as well as in the bulk via powder XRD (Fig 8).

**Table 3:** Slag H76-S39 SEM-EDS analyses, showing secondary calcite (1, 2, 3, 4), hematite (5, 6), and gypsum (7, 8).

*No.*	C	O	S	Ca	Fe
1	8.29	51.04	0.21	29.24	7.01
2	11.98	53.28	0.08	32.62	0.12
3	4.16	39.15	0.06	52.62	2.66
4	10.13	47.72	0.05	40.51	0.31
5	1.12	37.87	0.46	2.22	47.61
6	2.28	33.91	0.67	2.96	52.96
7	1.52	53.85	22.62	20.18	1.15
8	1.75	42.44	24.58	24.77	4.93

The calcite in the void was either nested (1) or lined (2, 3, 4). These minerals were confirmed to be present in the bulk powder XRD analysis. Complete EDS datasets are available in S2 Appendix.

Calcite and gypsum frequently form as secondary precipitates in slag voids and pores through the weathering of pre-existing calcareous and sulfidic materials [32,34,40,133]. Metallurgical copper slags are rich in Ca-bearing minerals such as pyroxenes (hedenbergite (CaFeSi_2_O_6_), in addition to primary calcite, gypsum, dolomite (CaMg(CO_3_)_2_), and gangue and added fluxes [26,40,56,66,102,106,109]. Similarly, copper slags contain sulfate ions from sulfur-bearing ores such as chalcopyrite (CuFeS_2_), bornite (Cu_5_FeS_4_), chalcocite (Cu_2_S), pyrrhotite (Fe_1-x_S), and pyrite (FeS_2_), in addition to entrapped matte droplets [29,32,109]. These minerals and phases would have contributed to the reprecipitation of calcite and gypsum within pores and voids. As discussed in greater detail elsewhere [32,39,113,134], dissolved Ca^2+^ ions in solution, from CO_2_ in soil and the atmosphere, which dissolve in water, form carbonic acid (H_2_CO_3_), bicarbonate (${HCO}_{\text{3}}^{-}$), and carbonate ions ${CO}_{\text{3}}^{\text{2}-}$, leading to neutral–alkaline conditions, with the latter reacting with the former to form calcite (Equation 3). These same Ca^2+^ ions, when saturated in solution with SO_4_^2-^ ions from oxidized and weathered sulfide minerals, lead to secondary gypsum precipitates (Equation 4).


$${Ca}^{\text{2}+}(aq)+{CO}_{\text{3}}^{\text{2}-}(aq)\to {CaCO}_{\text{3}}(s)$$ (3)

$${Ca}^{\text{2}+}(aq)+{SO}_{\text{4}}^{\text{2}-}(aq)+{\text{2}H}_{\text{2}}O(l)\to {CaSO}_{\text{4}}\cdot {\text{2}H}_{\text{2}}O(s)$$ (4)

These minerals commonly nucleate in Ca-containing copper slags, where an increase in surface area creates favorable microenvironments for crystal growth. The voids, which act as conduits for the flow and transport of water, provide these microenvironments [32,34,39,40,119,133,135]. X-ray diffraction also identified iron and calcium iron silicates such as fayalite and hedenbergite (CaFeSi_2_O_6_), oxides such as quartz and possibly cristobalite (SiO_2_), hematite and magnetite and iron oxide hydroxides, iron arsenate hydrates such as scorodite/parascorodite (FeAsO_4_·2H_2_O), iron sulfides like pyrrhotite (Fe_1-x_S) and pyrite (FeS_2_), with some of the latter being As-bearing, in addition to arsenopyrite (FeAsS), and löellingite (FeAs_2_). These minerals are typical of archaeological slags and those derived from modern flash smelters [26,29,32,40,56,100,102,109].

**Fig 9:**
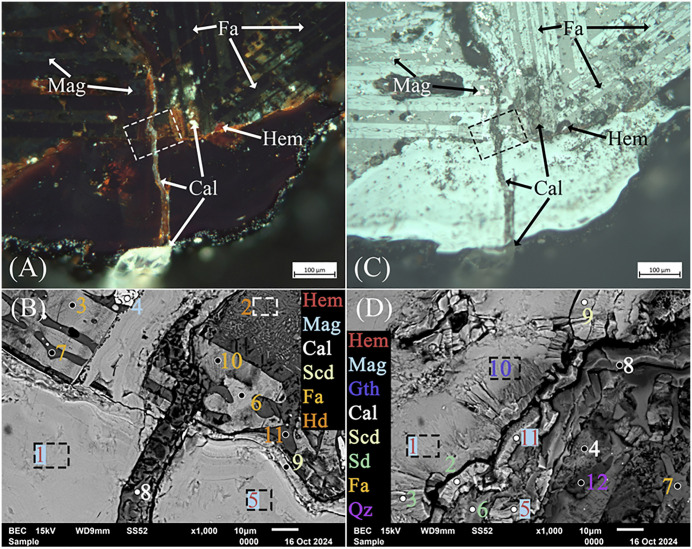
Optical images in dark- (A) and light-field (C) of the ‘Top’ of the large pore in slag H76-S39 showing lined calcite (Cal) on the inner surface and within cracks and the slag matrix. Numerous fayalite (Fa) lathes are also visible, in addition to isotropic magnetite (Mag) between them, interspersed within the pore lining with hematite (Hem). The white and black outlined boxes in (A) and (C) correspond to the SEM image and analysis locations for (B) and (D), respectively. (B) Backscatter image of the pore lining corresponding to the ‘Top’. Imaging and EDS analyses of the pore revealed the presence of calcite (Cal), fayalite (Fa) lathes in parallel, magnetite (Mag), hematite (Hem), scorodite (Scd), and hedenbergite (Hd). The outlined boxes represent analysis regions rather than points. Also shown is a crack/conduit filled with calcite and hematite precipitates that penetrated the pore’s Fe-oxide lining. (D) Backscatter image and EDS analyses of the ‘Bottom’ lining of the pore, showing hematite, magnetite, geothite (Gth), calcite, scorodite, siderite (Sd), fayalite, and quartz (Qz). The lining of the pore was intact, except for cracks where concentrations of calcite and hematite were found. Mineral abbreviations are from Warr (2021) [132].

**Fig 10:**
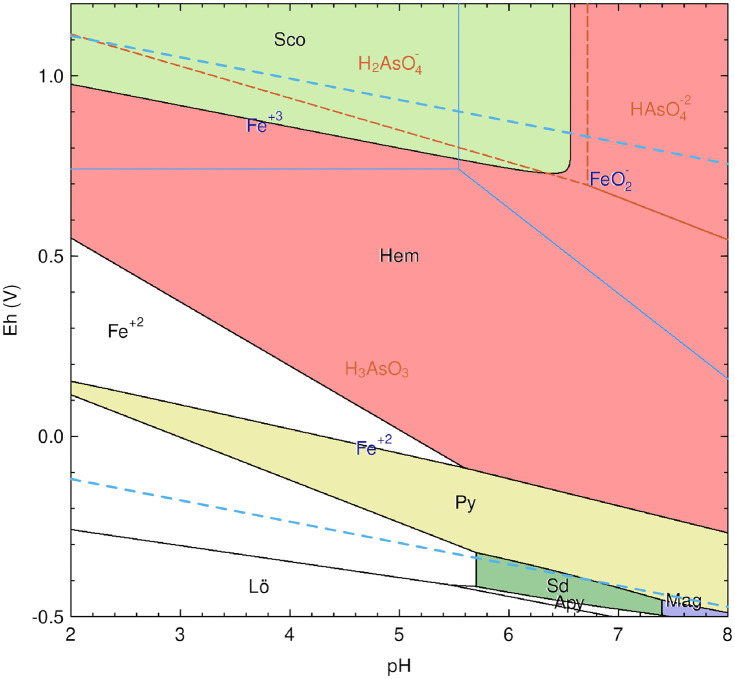
Simplified Pourbaix diagrams of the As-Fe-Si-C-H_2_O system for slag H76-S39 showing stability fields for scorodite (Sco) and siderite (Sd) at 25°C and 1.4 ATM. Mineral and aqueous species stability regions are outlined in black and blue, respectively, with corresponding text color, and red dashed lines and text delineate arsenic. Soluble sulfur species are not shown, but are necessary for the calculation. Related stable minerals that may form at specific Eh-pH in each instance include hematite (Hem), pyrite (Py), magnetite (Mag), arsenopyrite (Apy), and löllingite (Lo). According to these calculations, scorodite precipitates at < 2 to ~ 6.5 pH, and ~ 1 to 0.74 Eh (volt) in Fe^3+^(aq) and ${H_{\text{2}}AsO}_{\text{4}}^{-}(aq)$. Activity for each component was set as follows: S = −7, Fe = −2, As = 0, Si = −2, and C = −3. The activity of Cl was calculated to be −1.26985 based on the soil’s salinity and ionic strength (IS), 0.05372177 (*see* S3 Appendix).


In addition, optical microscopy and EDS analyses of the ‘top’ and ‘bottom’ of pore lining showed a black and ruby-red border of stoichiometric magnetite and hematite, long dark skeletal lathes of fayalite, often arranged in parallel, and colorless calcite (Fig 9A and 9C). These contain significant concentrations of As, Ca, and Al, along with a contingent of minor impurities (Table 4). The outliers in this group are analyses 2 and 11 (Fig 9B and 9D), which correspond to regions with high Al content in the slag. The accompanying Ca likely corresponds to small feather-like mineralizations of hedenbergite (CaFeSi_2_O_6_) that formed due to rapid cooling [29,38,46,105,118,136,137]. Of note, analysis 9 is likely scorodite, but with noticeably low As, which could have crystallized from weathered As-bearing minerals such as arsenopyrite [119,138,139], dissolved pyrite [140], relic sulfide inclusions from partially reacted As-bearing sulfides, or dissolved As in the slag [32,102]. Corrosion of these minerals, as shown in the Eh-pH diagram (Fig 10), can create localized high acidity, allowing for the spontaneous formation of scorodite from Fe^3+^ and ${AsO}_{\text{4}}^{\text{3}-}$ at < 4 pH or stepwise from hydrated Fe^3+^ and As^5+^ ions, such as ${Fe(H}_{\text{2}}{O)}_{\text{6}}^{\text{3}+}$ –the octahedral form in aqueous solution– and $H_{\text{2}}{AsO}_{\text{4}}^{-}$ at pH ranging from 2–7 (Equations 5), and the oxidation of arsenopyrite (Equation 6), which, along with magnetite and other iron oxides, release Fe^3+^ ions into solution when weathered (Equation 6) [39,138–142]. Given the stability of slag and its relatively neutral pH, the simplified complexation reaction shown in Equation 7 represents a paragenetic sequence that leads to the formation of scorodite. Siderite, another secondary precipitate, is known to form between pH 6.5 and 7 in anoxic, carbonated environments, in association with chukanovite, a metastable iron hydroxycarbonate (Fe_2_(OH)_2_CO_3_) [81,96,97,143–145], which, together, are associated with magnetite, goethite, scorodite, and iron sulfides such as pyrite [80,143,144,146]. These minerals are also shown in the diagram, and their proportions or presence/absence in the slag would depend on localized conditions. The association of these minerals is not unexpected, as Fe oxides and hydroxides, sulfates, carbonates, pyroxenes, and arsenate minerals are known to precipitate together in the pores of weathered copper slags [32,39,119,138,139,147–149]. Accordingly, these minerals, in addition to aluminum oxide (Al_2_O_3_), iron carbide (Fe_5_C_2_), löllingite (FeAs_2_), iron and calcium arsenate (FeAsO_4_), and parascorodite, were identified in the XRD spectra. Goethite was not present in the spectra, either due to its low concentration in the slag or because its peaks had been obscured by amorphous material.

**Table 4:** SEM-EDS analyses of the large, irregularly shaped pore lining in slag H76-S39.

*No./Top*	C	O	Si	Ca	Fe	As	*No./Bottom*	C	O	Si	Ca	Fe	As
1	0	37.57	1.9	0.41	51.35	5.71	1	0	37.16	1.71	0.4	57.78	1.07
2	0	35.21	29.39	5.97	16.17	1.95	2	7.42	26.92	2.98	1.8	55.75	2.5
3	0	33.48	18.36	0.66	43.39	2.92	3	12.01	29.09	1.22	0.85	52.23	1.18
4	0	35.81	3.59	0.69	53.03	5.06	4	15.37	50.07	11.23	19.59	1.27	0.84
5	0	37.18	1.56	0.35	49.99	7.54	5	9.44	40.92	5.69	0.81	39.11	2.25
6	0.6	35.57	6.01	0.52	53.27	2.57	6	12.1	52.77	3.23	28.24	0.77	1.52
7	0	34.29	17.47	0.49	43.82	3.03	7	6.07	36.39	26.89	6.18	10.53	1.59
8	9.89	47.93	0.61	38.36	1.55	0.62	8	12.63	40.35	1.3	41.82	1.52	1.2
9	0.62	34.73	5.15	1.2	43.02	12.93	9	5.13	31.53	1.64	0.59	55.19	3.82
10	0.78	35.78	17.69	0.44	41.56	2.64	10	0.65	27.49	1.95	0.87	64.15	3.01
11	0	42.26	33.28	2.53	8.1	0.98	11	3.05	10.71	0.97	3.93	75.3	3.91
–	–	–	–	–	–	–	12	2.1	51.7	43.64	0.3	0.74	0.27

The locations ‘Top’ and ‘Bottom’ correspond to opposite ends of the pore at its widest point. Analyses ‘Top’ showed high As impurities throughout, with most conforming to magnetite or hematite (1, 5), hedenbergite (2, 11), fayalite (3, 6, 7, 10), magnetite (4), calcite (8), and scorodite (9). Analyses ‘Bottom’ showed less As but far more C, with magnetite or hematite (1, 5, and possibly goethite 11), siderite (2, 3, 6), calcite (4, 8), scorodite or an iron arsenate hydrate (9), goethite (10), quartz and possibly cristobalite (12), fayalite or an iron-aluminum-silicate (7). XRD analyses confirmed the presence of these minerals/phases in the slag’s bulk, in addition to aluminum oxide, iron carbide (Fe_5_C_2_), löllingite (FeAs_2_), iron (FeAsO_4_), calcium arsenate, and parascorodite (chemically equivalent to scorodite). Goethite was not confirmed via XRD. Complete EDS datasets are available in S2 Appendix.

$${Fe}^{\text{3}+}(aq)+{AsO}_{\text{4}}^{\text{3}-}(aq)+{\text{2}H}_{\text{2}}O(l)\to {FeAsO}_{\text{4}}\cdot {\text{2}H}_{\text{2}}O(s)$$ (5)

$$\begin{array}{c} 4FeAsS+{\text{14}O}_{\text{2}}+{\text{12}H}_{\text{2}}O\to {4FeAsO}_{\text{4}}\cdot {\text{8}H}_{\text{2}}O+{4SO}_{\text{4}}^{\text{2}-}+{\text{8}H}^{+} \end{array}$$ (6)

$${Fe(H}_{\text{2}}{O)}_{\text{6}}^{\text{3}+}(aq)+{\text{4}H}_{\text{2}}{AsO}_{\text{4}}^{-}(aq)\to {Fe(H}_{\text{2}}{O)}_{\text{2}}{(H}_{\text{2}}{AsO}_{\text{4}}{)}_{\text{4}}^{-}(aq)+{\text{4}H}_{\text{2}}O(l)$$ (7)

**Fig 11:**
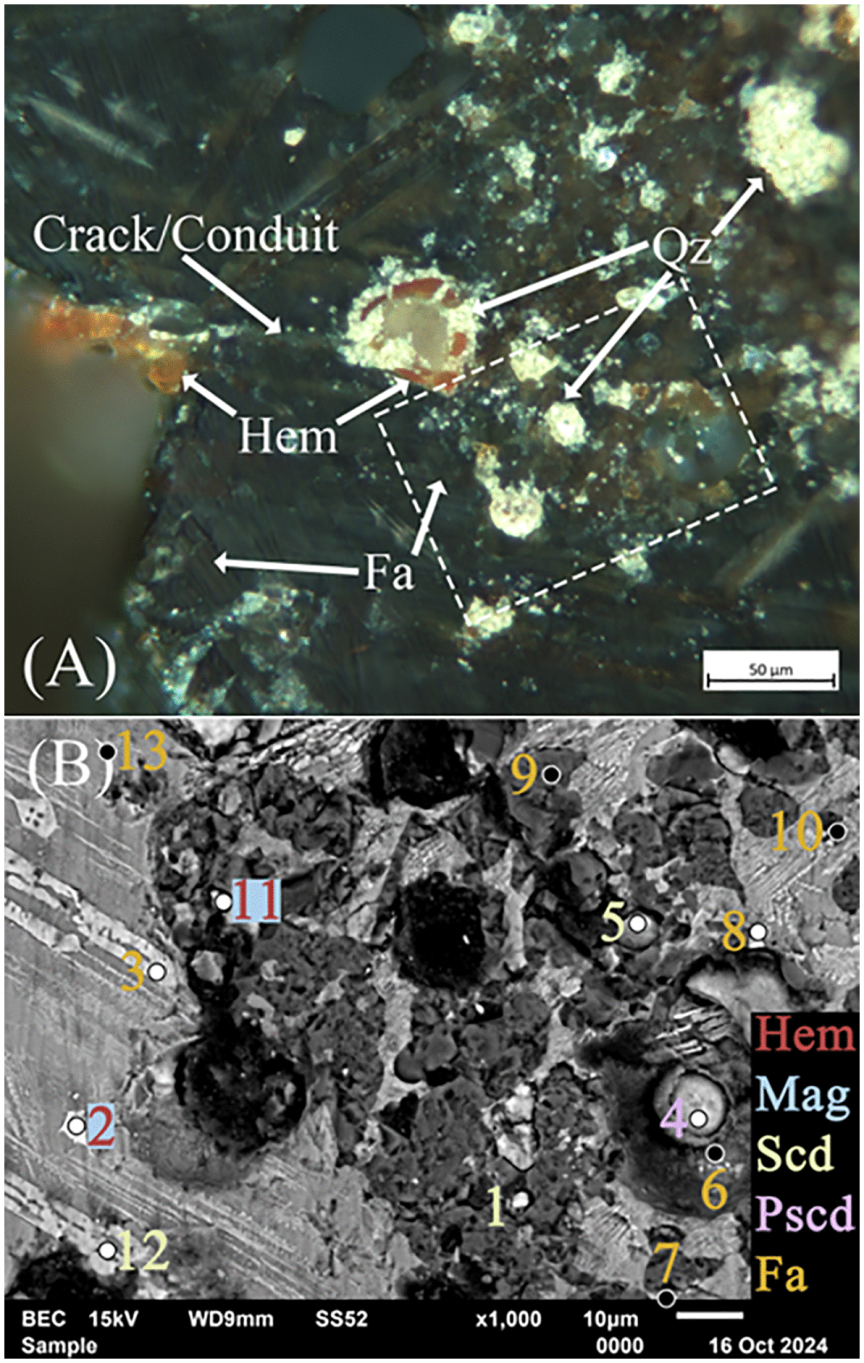
(A) Optical dark-field image and EDS analyses of the As-rich top region of slag H76-S39 with primary fayalite (Fa), secondary hematite (Hem), and quartz (Qz). A crack or conduit connects the surface to the inner pores. The box outlined in white corresponds to the SEM image and analysis. (B) Backscatter image of secondary scorodite (Scd), parascorodite (Pscd), and numerous small quartz (Qz) crystal precipitates. Mineral abbreviations are from Warr (2021) [132].

Finally, the high concentrations of As, especially for analyses 9 ‘Top’ and 1, 4, 5, and 11 near the surface of the slag, require explanation (Fig 11A). Table 5 presents analyses of the area immediately below the observed surface corrosion, identified by XRF as As-rich. Stoichiometrically, analyses 1, 5, and 11 are closest to scorodite, with 4 having As percentages consistent with parascorodite (Fig 11B). Both are secondary precipitates formed from weathered As-bearing minerals at ambient temperatures, pressures, and < 3.5 pH [138,139,150]. Furthermore, given the availability of As and the multitude of impurities in the analyses, much of the discovered scorodite may be amorphous ferric arsenates (AFA), also referred to as hydrous ferric arsenates (HFA), which form as an intermediary step to crystalline scorodite or parascorodite, but only at pH < 3 [119,138,139]. These arsenates quickly transition to thermodynamically stable scorodite or parascorodite [138], but can also slowly grow in neutral to alkaline pH environments [119,139,151].

**Table 5:** SEM-EDS analyses of the area below the As-rich top identified by XRF in slag H76-S39.

*No.*	C	O	Si	Ca	Fe	As
1	4.86	28.57	28.11	4.73	13.31	18.98
2	4.14	22.4	10.5	1.46	55.74	1.49
3	10.69	32.66	20.32	0.96	28.35	1.66
4	3.64	13.89	8.07	8.57	28.07	34.36
5	14.18	33.34	5.03	6.87	17.86	20.2
6	2.08	34.36	23.39	1.41	30.92	2.61
7	4.16	37.19	27.43	6.61	17.71	0.79
8	15.57	39.19	20.63	1.55	11.63	1.1
9	11.9	39.33	34.14	0.5	8.95	0.2
10	8.86	36.81	26.59	5.43	16.1	1.14
11	14.35	33.04	10.94	5.59	17.44	14.7
12	6.45	33.93	12.71	1.18	38.72	4.47
13	9.37	29.24	24.39	3.92	23.54	2.73

The amount of As varies from less than a percent to just above 35 wt.%. The implication of high As levels in this region is that the resultant mineralogy is derived from weathering rather than pyrometallurgy. The SEM-EDS analyses align with scorodite for samples 1, 5, and 12, and with parascorodite for sample 4. Analyses also revealed the presence of hematite or magnetite (2, 11) and fayalite (3, 6, 7–10, 13). Complete EDS datasets are available in S2 Appendix.

In addition to ferric arsenates and scorodite minerals, what is evident from these analyses is the appreciable amounts of As, regardless of mineralogy. The EDS data, in agreement with XRF, showed a more significant proportion of As-bearing phases residing just below the surface of the slag in the altered regions. And while XRD confirmed the presence of As-bearing secondary precipitates, it also showed löellengite or FeAs_2_. Iron diarsenide can form naturally as a mineral through hydrothermal reactions in geological settings at temperatures ranging from 400 to 500°C in pyrrhotite and serpentinite veins [152], or during high-temperature pyrometallurgical processing between 900 and 1200°C under reducing conditions, followed by the thermal decomposition of arsenopyrite and other iron arsenides, such as FeAs [58]. Processually, where high temperatures and chemistries persisted to form Fe_2_As and FeAs during smelting, the latter likely resulted in FeAs_2_ upon cooling at temperatures between 510–690°C, as solid FeAs reacted with highly active As from the melt [58,86,153]. These anthropogenically created iron arsenides could then, according to the Eh-pH diagram, oxidize to scorodite [119,138,139,141], following Equation 8 at low pH. Notably, at pH above ~ 4.7, arsenic mobilization would be limited.


$$\begin{array}{c} {FeAs}_{\text{2}}(s)+{\text{1}\text{4}Fe}^{\text{3}+}(aq)+{\text{1}\text{2}H}_{\text{2}}O(l)\to [{\text{2}FeAsO}_{\text{4}}\cdot {\text{2}H}_{\text{2}}O(s)]+{\text{1}\text{3}Fe}^{\text{2}+}(aq)+{\text{1}\text{6}H}^{+}(aq) \end{array}$$ (8)

Understanding the role of As during smelting and its omnipresence in the slag is of the utmost importance to archaeometallurgists and modern material scientists alike. What is clear from the studied slag is that while the proportions of As necessary to form iron arsenides existed, the conditions were insufficient to form large homogeneous speiss phases. Prior studies have, however, identified small amounts of noncontiguous speiss prills [49,52,54,154].

The likely explanation for the observed high As in these slags is that, during cooling, it was incorporated into the fayalite glassy silicate matrix and, in some instances, became encapsulated, forming intermetallic phases with Fe [138]. Arsenic can also be distributed to the fayalite phase and glassy silicate matrix through the substitution of ${SiO}_{\text{4}}^{\text{4}-}$ for ${AsO}_{\text{4}}^{\text{3}-}$ tetrahedral groups during the smelting of ore-containing olivines in an oxygen-rich atmosphere [138,155,156]. Notably, olivines are prevalent in Iranian copper ores [26,102,157,158]. As for any serendipitously discovered speiss prills, these explanations would account for their scarcity and location in the slag (*see* S5 Appendix for a more detailed explanation). Tangentially related to speiss, arsenic could have also been distributed through the alteration of primary arsenic minerals, phases, and As-bearing fluids, resulting in ferric arsenates, hydrous ferric oxide (HFO), and minerals like scorodite [119,138,139].

**Fig 12:**
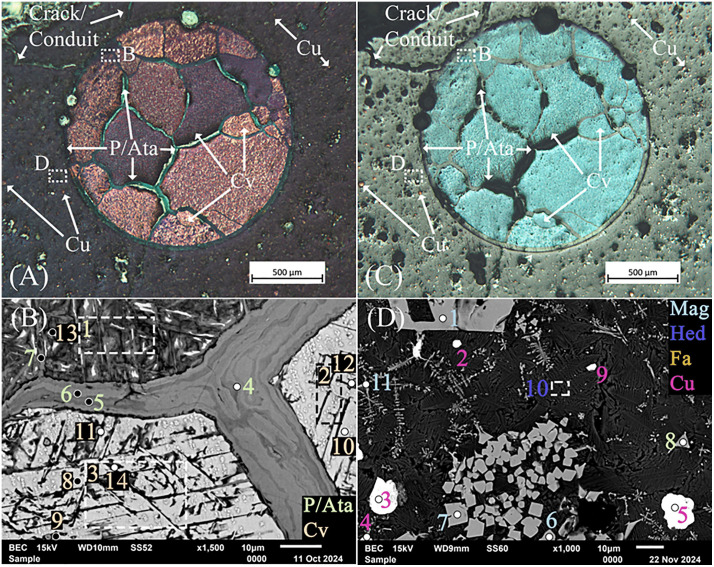
Optical images in dark- (A) and light-field (C) of the sectioned high-density droplet in slag H76-S37A showing covellite (Cv) in a distinctive indigo-blue color and Widmanstätten-like structure. The white outlined boxes in (A) and (C) correspond to the SEM image and analysis locations for (B) and (D), respectively. Also shown are the extensive intergranular and transgranular atacamite (Ata) or paratacamite (Pata) secondary precipitates surrounding and pervading the droplet. Numerous intact Cu droplets are also visible, having not been connected to the surface through cracks or conduits. The cracks or conduits extend from the surface to the droplet. (D) Backscatter image, with location shown in (A) of variously oriented leached covellite grains with intergranular, transgranular, and Widmanstätten-like corrosion patterns. The outlined boxes represent analysis regions rather than points in (B) and (D). (D) Backscatter image of the slag, delineated in (D), showing smaller copper (Cu) droplets, magnetite (Mag), and feather-like hedenbergite (Hed) within the fayalite (Fa) matrix. Mineral abbreviations are from Warr (2021) [132].

After sectioning slag H76-S37A at the prescribed coordinates, a large metallic teal droplet and multiple smaller Cu-colored droplets were exposed; the smaller ones were unresolvable due to their small size. Optical imaging of the large droplet showed distinct teal and white crosshatching in light-field and a metallic luster in dark-field (Fig 12A and 12C), which we could not attribute to any known slag minerals, phases, or precipitates [32,159–164]. Encircling and between the grains are emerald-green colored phases identified as atacamite and later confirmed by EDS and XRD. The droplet exhibited a Widmanstätten-like structure. In this instance, given the extensive intergranular and transgranular corrosion throughout (Fig 12B and 12D), the pattern is undoubtedly the result of leaching and the formation of secondary precipitates along slip lines and planes [161,165,166]. Energy-dispersive X-ray spectroscopy of the intergranular, transgranular, and slip line alterations consistently showed atacamite or paratacamite (Table 6), with the latter being the more thermodynamically stable polymorph at high chloride concentrations [84,167,168]. The grains themselves were also consistently identified as copper sulfides by EDS with varying ranges of Cu, which, having been leached, predominantly resulted in ${CuCl}_{\text{2}}^{-}$ ions in solution while enriching the overall proportion of S [159,169–172]. Small copper droplets, magnetite, atacamite or paratacamite, and fayalite with possible Al and Ca substitutions were also found adjacent to the larger droplet in the slag’s matrix.

**Table 6:** SEM-EDS of the teal droplet in slag H76-S37A2.

*No./Inner*	C	O	Si	S	Cl	Fe	Cu	As	Se	*No./Outer*	C	O	Si	S	Cl	Fe	Cu	As	Se
1	1.65	25.38	4.27	4.46	5.97	0.96	50.04	1.84	1.97	1	0.62	22.83	0.29	0.08	0.20	67.66	1.00	0.73	3.06
2	0.52	4.45	0.40	26.85	0.60	0.52	63.74	2.22	0.29	2	3.68	2.85	1.31	0.09	0.44	2.85	81.88	1.30	4.48
3	1.31	9.93	1.43	22.81	0.43	1.08	58.09	0.78	3.17	3	1.35	1.87	0.26	0.19	0.76	1.58	89.39	1.93	2.27
4	0.73	20.30	0.26	0.14	15.70	0.78	54.48	2.12	4.70	4	2.57	2.32	0.32	0.12	1.22	2.04	87.58	1.40	2.14
5	0.76	17.47	1.00	2.38	13.16	0.44	59.64	1.72	2.31	5	0.96	1.76	0.27	0.13	0.83	1.76	84.84	2.58	6.44
6	0.44	18.48	0.41	0.33	15.90	1.13	59.25	0.18	2.73	6	0.11	23.98	0.19	0.10	0.06	69.50	0.82	0.54	2.40
7	0.39	17.50	1.15	0.57	14.59	0.83	57.24	2.18	4.36	7	0.23	24.14	0.31	0.04	0.06	67.04	0.87	1.82	2.64
8	0.68	3.26	1.75	22.04	0.74	1.40	64.70	1.55	2.71	8	1.09	26.13	6.85	0.11	0.60	53.54	2.35	2.37	1.95
9	0.66	6.75	1.39	22.40	0.80	0.51	63.06	0.96	1.96	9	3.74	2.94	0.89	1.28	0.59	2.57	83.31	1.16	2.70
10	0.23	0.51	0.13	28.97	0.22	0.26	62.83	1.76	4.97	10	0.46	29.80	28.70	0.03	0.66	15.91	1.34	1.17	0.45
11	0.71	1.78	0.44	28.98	0.34	0.47	62.87	0.97	2.90	11	0.07	23.55	6.15	0.06	0.19	60.17	0.72	2.09	2.44
12	0.92	1.98	0.39	27.61	0.29	0.45	63.05	0.43	4.48	–	–	–	–	–	–	–	–	–	–
13	0.13	10.41	1.80	19.14	2.45	0.53	56.82	0.86	4.49	–	–	–	–	–	–	–	–	–	–
14	0.99	6.73	1.82	22.16	0.72	0.48	61.88	1.12	1.28	–	–	–	–	–	–	–	–	–	–

Analyses ‘Inner’ showed atacamite or paratacamite (4, 5, 6, 7) and covellite (2, 3, 8, 9, 10, 11, 12, 13, 14). Analysis 1 is also covellite, but since it was taken across a broad region that includes the initial covellite grain and slip line corrosion, it also contains Cl. Analyses ‘Outer’ also showed phases with more impurities, including magnetite (1, 6, 7, 8, 11) with minor Se, copper (2, 3, 4, 5, 9) with C, O, and Se, hedenbergite (10), and fayalite (11) with Na. XRD analysis confirmed the presence of these minerals/phases in the slag’s bulk in addition to aluminum oxide (Al_2_O_3_), hedenbergite, arsenolite (As_2_O_3_), augite (CaMg_0.75_Fe_0.25_Si_2_O_6_), quartz, and sodium sulfide (Na_2_S). Complete EDS datasets are available in S2 Appendix.

**Fig 13:**
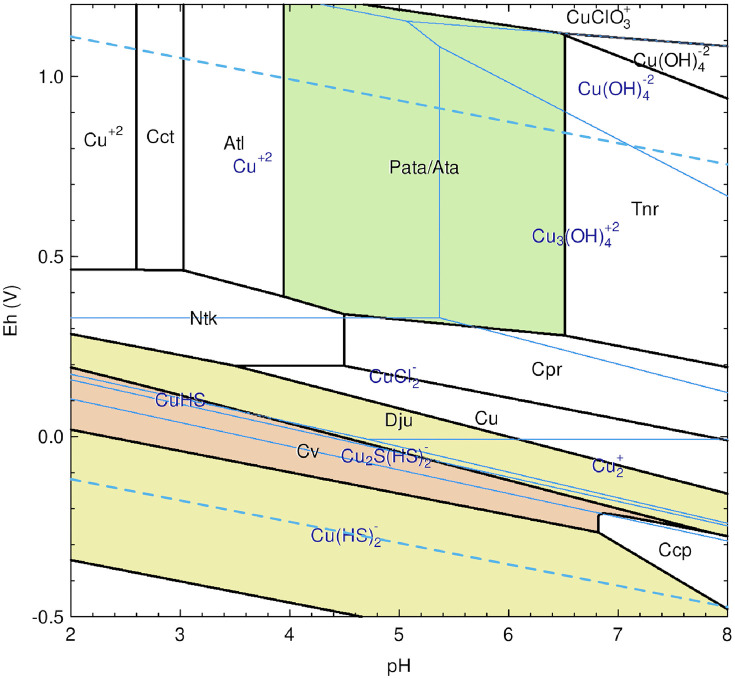
Simplified Pourbaix diagram calculation of the Cu-Fe-S-Cl-H_2_O system for slags H76-S37A and -S45B showing stability fields for (par)atacamite (Pata/Ata) at 25°C and 1.4 ATM. Mineral and aqueous species stability regions are outlined in black and blue, respectively, with corresponding text color. Soluble sulfur species are not shown, but are necessary for the calculation. Related stable minerals that may form at specific Eh-pH include antlerite (Atl), chalcanthite (Cct), tenorite (Tnr), cuprite (Cpr), nantokite (Nik), djurleite (Dju), covellite (Cv), and chalcopyrite (Ccp). According to these calculations, (par)atacamite precipitates at ~ 4 to 6.4 pH and ~ 0.3 to 1.2 Eh (volt) in the presence of ${CuCl}_{\text{2}}^{-}(aq)$ and oxygen, resulting in the formation of Cu^+^(aq). Activity for each component was set as follows: S = −2, Cu = 0, Fe = −15. Furthermore, the activity of Cl was calculated to be −1.26985 based on the soil’s salinity and ionic strength (IS), 0.05372177 (*see* S3 Appendix).

Metal-bearing sulfides are particularly susceptible to weathering under oxidizing conditions in both air and aqueous environments, releasing metallic elements into solution [32,40,173], as shown in the Eh-pH diagram (Fig 13). Slag H76-S37A is a unique example of this phenomenon, as Cu ions were slowly leached from the covellite droplet [169,172], resulting in surrounding and infiltrating atacamite or paratacamite precipitates, even along slip planes (Table 6 ‘Inner’). Based on these findings, a likely scenario for its structure and composition is that the copper sulfides formed during smelting, under low oxygen fugacity, or during cooling [26,30,37,40,59,102,109–111], were subsequently leached and corroded through two successive reactions. Beginning with chalcocite, or, more precisely, a comparable Cu_2_S phase formed during smelting [109,174], the sulfide would have been rapidly oxidized by Fe^3+^ ions at ambient temperatures, resulting in Cu-deficient “blue-remaining” covellite with hexagonal structure (Equation 9) [170,175–181]. Following this transformation, if the droplet were not already monoclinic Cu_2_S or white metal, leaching would produce Cu²⁺ ions and elemental S^0^ [159,169–172]. Small copper droplets, magnetite, atacamite or paratacamite, and fayalite with possible Al and Ca substitutions were also found adjacent to the larger droplet in the slag’s matrix.

The first oxidation transformation (Equation 9) is controlled by the diffusion of the oxidant on the mineral surface, and the second (Equation 10), by chemical and/or electrochemical reactions [159,169,170,172,175,182–184]. During leaching, Fe^3+^ ions, released from efflorescent salts and weathered minerals such as magnetite and goethite [34,80], react, releasing Cu^2+^ ions and shifting the redox potential towards more reducing conditions [83,175,185]. If oxygen remained, Fe^2+^ could then oxidize to Fe^3+^ [30,40,186,187], contributing to the continued dissolution of chalcocite and covellite. Availability of Fe^3+^ could have also come from the oxidation of fayalite to hematite and silica [67,188], a reaction between chalcocite and fayalite [189], or by arsenate oxidants and selective sulfide-reduction-volatilization [190].


$${Cu}_{\text{2}}S(s)+{\text{2}Fe}^{\text{3}+}(aq)\to {Cu}^{\text{2}+}(aq)+{\text{2}Fe}^{\text{2}+}(aq)+CuS(s)$$ (9)

$$CuS(s)+{\text{2}Fe}^{\text{3}+}(aq)\to {Cu}^{\text{2}+}(aq)+{\text{2}Fe}^{\text{2}+}(aq)+S^{\text{0}}(s)$$ (10)

In these reactions, Cl⁻ ions, from salts such as NaCl in solution, enhance the corrosion kinetics such that [182], if it is absent, a layer of dense amorphous elemental S can form on the mineral surface, hindering diffusion and slowing the reaction [170,175,182]; however, if present, they promote the formation of a porous crystalline sulfur layer, allowing the leachant to penetrate and react with the underlying copper sulfide phases [159,170,171,175,182,191]. Chloride ions also stabilize Cu⁺ cations by forming ${CuCl}_{\text{2}}^{-}$, facilitating the dissolution of Cu^2+^ ions into solution while avoiding the intermediary step of oxidizing Cu^+^ [172,175]. Further accelerated leaching occurs from increased proportions of chlorides (Equations 11 and 12) [167,175,192], which, in this slag, resulted in CuS and soluble ${CuCl}_{\text{2}}^{-}$, and potentially elemental S and ${CuCl}_{\text{2}}^{-}$ [159,169,175,182,191]. Eventually, the remaining Cu would be leached from the covellite for as long as chlorides and water were available for the reaction, leaving elemental S behind.


$${Cu}_{\text{2}}S(s)+{\text{2}Cl}^{-}(aq)+H^{+}(aq)+{\frac{\text{1}}{\text{4}}O}_{\text{2}}(g)\to {CuCl}_{\text{2}}^{-}(aq)+{\frac{\text{1}}{\text{2}}H}_{\text{2}}O(l)+CuS(s)$$ (11)

$$CuS(s)+{\text{2}Cl}^{-}(aq)+H^{+}(aq)+{\frac{\text{1}}{\text{4}}O}_{\text{2}}(g)\to {CuCl}_{\text{2}}^{-}(aq)+{\frac{\text{1}}{\text{2}}H}_{\text{2}}O(l)+S^{\text{0}}(s)$$ (12)

These combined reactions likely explain the droplet’s slow inwardly progressing transformation through narrow surface-connected cracks/conduits, as well as intergranular and transgranular leaching across the slight potential differences between grains, grain boundaries, and slip planes (Fig 12B) [80,193–196]. Our extensive SEM-EDS investigation furthermore revealed the presence of several cracks/conduits throughout the slag, which often led to leached copper sulfide droplets, as shown in Fig 12A and 12C. Where infiltration channels exist, the leaching of Cu^2+^ ions was favored, and at increasing Cl^-^ concentrations and in the presence of S, intergranular leaching along high-angle grain boundaries was more probable [171,172,175,183,191,193]. The reactions are facilitated through the formation of large S crystals, which create porous, penetrable layers for the reactions to continue unabated [169,182]; however, when formed, basic salts such as atacamite and paratacamite would have slowed or halted the reaction [169], effectively freezing further leaching/corrosion and passivating the system.

Having leached Cu^2+^ and stabilized ${CuCl}_{\text{2}}^{-}$, the formation of atacamite could proceed according to Equation 13, as shown in the Pourbaix diagram (Fig 13). Notably, this is not the only pathway leading to copper oxychloride precipitates [167,192,197], but it is the most likely one, given the association of copper sulfides with high Cl^-^ concentrations, the latter being common in the form of sodium chloride (NaCl) in modern-day Semnan [76,198].

**Fig 14:**
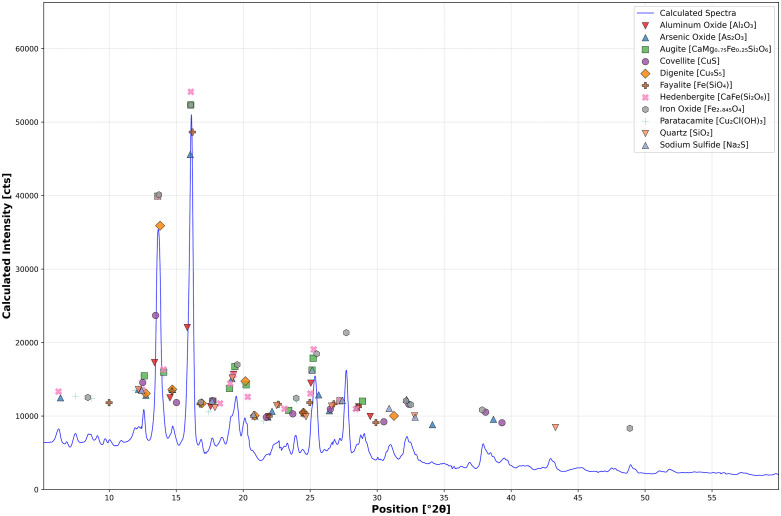
X-ray diffraction spectra of slag H76-S37A. Several of the detected phases and minerals were previously identified using optical microscopy and EDS, except for aluminum oxide, arsenic oxide, hedenbergite, digenite, augite, quartz, and sodium sulfide. The presence of digenite is likely evidence of the gradual leaching of Cu_2_S phases. Similar to the other studied slags, a large percentage consists of an amorphous phase, which obscures many of the mineral-defining peaks. Rietveld refinement was employed to determine the mineral composition of the slag based on the phases and minerals identified by optical microscopy and SEM-EDS.


In addition, of note, leached Cu^2+^ leads to a series of non-stochiometric copper sulfides, or polysulfides or intermediaries, such as djurleite, digenite, anilite (Cu₁.₇₅S), geerite (Cu₁.₆S), spionkopite (Cu₁.₄S), and yarrowite (Cu₁.₁S) [159,181]. Of these minerals, only covellite and digenite were confirmed via XRD, suggesting the discussed transformation of chalcocite into non-stochiometric copper sulfides between end-members Cu_2_S and Cu_2_S_2_ had occurred [181,199]. Aluminum oxide, hedenbergite, arsenolite (As_2_O_3_), augite (CaMg_0.75_Fe_0.25_Si_2_O_6_), quartz, and, possibly, sodium sulfide (Na_2_S) were also identified in the XRD spectra (Fig 14).

$${\text{3}CuCl}_{\text{2}}^{-}(aq)+{\frac{\text{3}}{\text{4}}O}_{\text{2}}(g)+{\frac{\text{3}}{\text{2}}H}_{\text{2}}O(l)\to {Cu}_{\text{2}}Cl(OH{)}_{\text{3}}(s)+{Cu}^{\text{2}+}(aq)+{\text{5}Cl}^{-}(aq)$$ (13)

**Fig 15:**
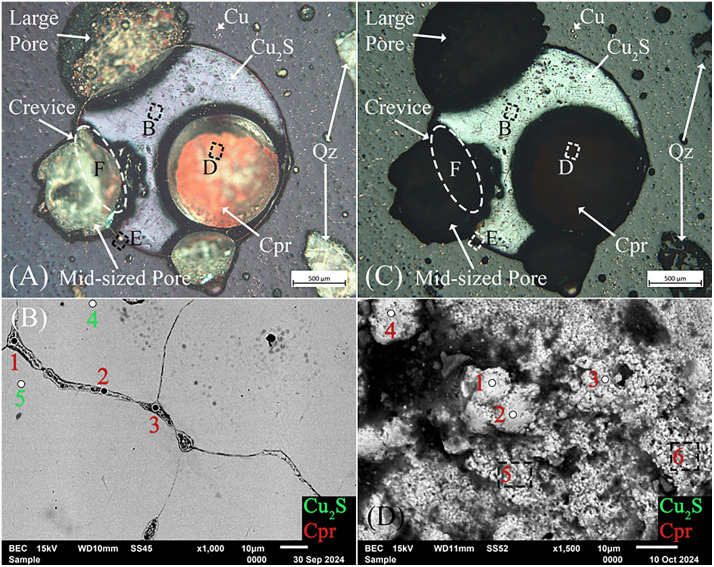
Optical dark- (A) and light-field (B) images of slag H76-S45B. The droplet is metallurgical Cu_2_S with fine Cu droplets and quartz (Qz) surrounding it. Notably, the large silver droplet also contains As, which contributes to its silvery appearance. The black and white outlined boxes in the optical images correspond to SEM-EDS images (B) and (D). Within the larger droplet is a void containing cuprite (Cpr), as shown in (A), (D), and between grains in (B). Also shown in the optical images are regions E and F, corresponding to the SEM-EDS images and analysis locations. Mineral abbreviations are from Warr (2021) [132].

**Fig 16:**
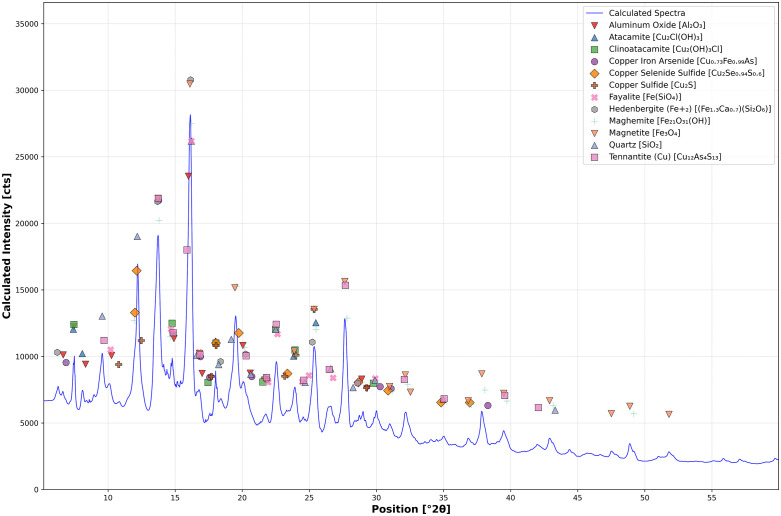
X-ray diffraction spectra of slag H76-S45B. Several of the detected phases and minerals were identified via optical microscopy and EDS, except for clinoatacamite, copper-iron arsenide, tennantite, magnetite, and hematite. Magnetite and hematite were prominently observed via optical microscopy elsewhere in the slag. Comparatively, cuprite was not detected, despite its assured existence as oxidation products within the Cu_2_S droplet. As before, the large percentage of amorphous phase obscured many of the peaks, requiring Rietveld refinement based on known phases and minerals.

A large droplet in slag H76-S45B was identified using XCT, sectioned, and subsequently studied using optical and scanning electron microscopy, as well as X-ray diffraction (XRD). Due to the droplet’s size and the fact that it was sectioned along the same axis as its scanned orientation, the droplet, its internal spherical void, and the surrounding pores aligned precisely with the layout observed in the CT scan. When polished, the droplet appeared silvery with Cu and quartz distributed near it within the slag matrix, and its void contained an internal lining of red mineralizations (Fig 15A and 15C). The void minerals exhibited intense X-ray attenuation compared to the droplet, suggesting that they contained metal-rich secondary precipitates. Optical microscopy and EDS analyses showed the droplet to be a silvery-white metal with appreciable concentrations of Se, As, low Fe (Table 7 ‘Inner’), and intergranular cuprite (Fig 15B and 15D). The void mineralizations stoichiometrically conformed to cuprite. X-ray diffraction confirmed the presence of Cu_2_S, in addition to: aluminum oxide, copper-iron-arsenide (Cu_0.73_Fe_0.99_As), tennantite-(Cu) (Cu_12_As_4_S_13_), copper-selenide-sulfide (Cu_2_S_0_._06_Se_0.94_), clinoatacamite (Cu_2_(OH)_3_Cl), atacamite, magnetite, maghemite (γ-Fe_2_O_3_), and fayalite (Fig 16). Several of these minerals appear in the Pourbaix diagram (Fig 13); however, since thermodynamic data is lacking for Cu-, Se-, and Fe-containing species, more representative stability regions could not be calculated for this slag.

**Table 7:** SEM-EDS of the large silvery droplet in slag S45B.

*No./Inner*	C	O	Si	S	Cl	Ca	Fe	Cu	As	Se	*No./Outer*	C	O	Si	S	Cl	Ca	Fe	Cu	As	Se
1	1.69	5.84	1.11	0.7	1	0.18	1.02	81.8	1.48	4.95	1	0.45	1.77	0.44	0.29	0.79	0.15	1.7	89.79	4.12	0.23
2	1.08	9.76	0.51	1.98	1.24	0.2	0.29	79.76	3.33	1.6	2	0	31.29	23.28	0	0.51	1.34	31.71	0.78	2.34	1.71
3	2.84	11.8	1.06	4.4	1	0.12	0.62	72.42	2.63	2.63	3	0	33.02	28.19	0	0.29	12.22	17.09	0.21	0.72	0.58
4	1.69	1.01	0.49	17.33	0.51	0.13	0.45	70.04	1.74	6.43	4	0.78	1.49	0.43	19.01	0.35	0.09	0.69	73.21	0	3.78
5	1.41	1.52	0.61	18.67	0.6	0.07	0.46	72.91	1.46	1.97	5	2.7	2.68	0.64	17.16	1	0.1	0.68	70.57	1.92	2.37
6	1.61	10.24	0.58	0.2	1.78	0.02	0.32	72.47	5.73	6.64	6	0.16	0.71	0.37	18.4	1	0.11	0.81	75.2	1.38	1.71
7	0.81	6.74	0.45	0.15	1.81	0.06	0.32	77.26	2.93	9.25	7	0	20.74	0.62	0.27	1.35	0.01	20.09	23.12	31.17	1.82
8	1.03	9.32	0.4	0.37	0.88	0	0.01	70.79	4.85	12.17	8	2.76	6.46	1.48	0.19	0.73	0.12	1.83	80.29	1.49	4.29
9	2.35	8.19	3.92	0.52	0.75	0.24	1.34	75.5	0	6.92	9	5.11	6.22	1.73	3.2	1.59	2.78	3.97	71.57	0.51	2.44
10	6.7	10.92	1.43	0.86	0.87	0.28	0.9	69.7	2.92	5.05	10	3.72	7.41	0.48	0.27	1.3	0.23	1.53	79.63	2.66	2.56
11	4.21	14.99	1.13	1.9	0.94	0.28	0.75	59.82	4.13	10.98	11	1.49	5.16	0.83	0.4	17.45	0.04	2.64	63.4	3.08	5.28
–	–	–	–	–	–	–	–	–	–	–	12	2.1	19.21	0.62	0.86	1.96	0.03	28.11	8.3	33.36	3.34
–	–	–	–	–	–	–	–	–	–	–	13	1.23	12.85	0.68	0.18	21.17	0.17	2.15	58.57	0	2.79
–	–	–	–	–	–	–	–	–	–	–	1	0.45	1.77	0.44	0.29	0.79	0.15	1.7	89.79	4.12	0.23

Analyses ‘Inner’ correspond with cuprite (1–3) between grains of Cu_2_S (4, 5). Cuprite was also found within the droplet’s internal pore (6–11). Analyses ‘Outer’ showed cuprite (1, 8–10), fayalite (2), hedenbergite (3), chalcocite (4–6), agreeing with the ‘Inner’ analyses, atacamite/paratacamite (11, 13), and indeterminable copper-iron-arsenates or -arsenites (7, 12). XRD analysis also detected aluminum oxide, copper-iron-arsenide (Cu_0.73_Fe_0.99_As), tennantite-(Cu) (Cu_12_As_4_S_13_), copper-selenide-sulfide (Cu_2_S_0_._06_Se_0.94_), clinoatacamite (Cu_2_(OH)_3_Cl), atacamite, magnetite, and maghemite (γ-Fe_2_O_3_), but no cuprite. Complete EDS datasets are available in S2 Appendix.

**Fig 17:**
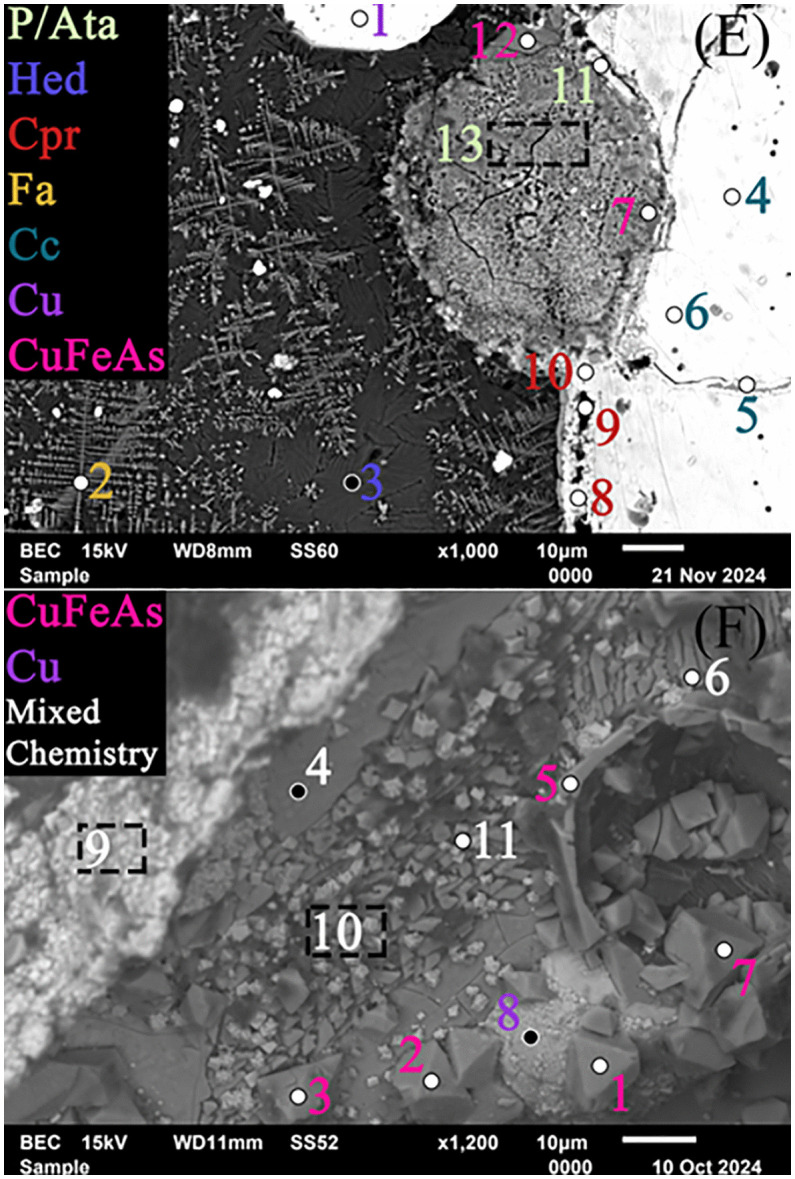
SEM-EDS images and analyses of slag H76-S45B regions (E) and (F) as shown in optical photos (A) and (C). Analyses (E) confirm that the droplet, as previously observed, comprises metallurgical Cu_2_S or chalcocite (Cc) and cuprite (Cpr) with high concentrations of Se and As. Immediately next to the droplet, between the slag matrix and the droplet, lies an atacamite/paratacamite (P/Ata) precipitate, within which are two copper-iron-arsenides (CuFeAs). Also shown are a Cu droplet, hedenbergite (Hed), and fayalite (Fa). SEM-EDS image (F) shows several tetragonal copper-iron-arsenide crystals. These reside within a crevice beside the large droplet and the ‘Mid-size’ pore. This is a unique microenvironment where these crystals and pure Cu had precipitated. Mineral abbreviations are from Warr (2021) [132].

Notably, the analyses of the droplet and surrounding phases reveal significant concentrations of As, Se, or both, which, together, could have imparted a silvery coloration to the already white-metal-colored droplet [200,201]. The droplet is a curiosity, given its similarity to modern-day copper matte white metal in copper converting, which contains ~ 75% Cu, with Se tending to distribute to it under reducing conditions [107,202–204]. Arsenic, on the other hand, tends to be distributed to Cu [205,206], although it can also be found in the white metal with increasing concentrations of As from primary ores [200,207,208]. Furthermore, archaeometallurgical studies of As-containing Cu have shown that silvery surface coloration, resulting from inverse segregation under non-equilibrium conditions, can occur through the formation of a γ-phase (Cu_3_As) layer [209–211]; however, even incomplete layers and low As concentrations can contribute to silvered appearances. In brief, while chalcocite is typically dark blue or black [30,32,105], pyrometallurgical Cu_2_S is white, and when alloyed with As and Se, it can result in a silvery color [59,102,212]. Abutting the droplet immediately beside the mid-sized pore is a minor atacamite/paratacamite secondary mineralization, and within it, shown in Table 7 ‘Outer’, analyses 7 and 12, are copper-iron-arsenates or -arsenites (Fig 17E and 17F).

Within the largest of the three pores surrounding the droplet are cuprite and calcite mineralizations, which contain copper-carbon-oxides or possibly Cu carbonates. These minerals can sequester Cu and inhibit further dissolution of metal-bearing phases, forming minerals like malachite and azurite [40,213,214]. The difference between the cuprite in the ‘Inner’ and ‘Large’ void and pore, respectively, seems to be in the contiguousness of the precipitate, with the void mineralization being an uninterrupted layer capable of attenuating X-rays. In contrast, those in the pore are sporadically distributed among less dense minerals such as calcite. Furthermore, as seen in Fig 15A and 15C, the pore resides between the droplet and the slag matrix; however, the void is entirely enclosed within the droplet, thus creating a microenvironment with limited ion migration.

As for the copper-iron-arsenide, SEM-EDS clearly showed several truncated tetragonal (di)pyramidal crystals in the ‘Mid-sized’ pore and adjacent crevice, which are not pyrometallurgical in origin; rather, they are secondary precipitates borne of a unique microenvironment, underscoring the fact that weathering and the dissolution and recrystallization of phases and minerals is commonplace in ancient, historical, leading to minerals typically uncharacterized in modern slags [39–41,133,149,215–217]. X-ray diffraction identified tetragonal copper-iron-arsenide, which may be related to those found in the crevice; however, this is uncertain, as they were not targeted separately. The high concentration of Se is also of interest, as it is prevalent in almost every EDS analysis (Table 8), likely due to its known distribution to white metal during the smelting process.

**Table 8:** SEM-EDS analyses of the mineralizations within the crevice between the droplet and the ‘Mid-sized’ pore in slag H78-S45B.

*No.*	C	O	Fe	Cu	As	Se
1	1.77	1.92	42.15	15.69	32.28	5.24
2	3.51	3.93	39.04	19.82	28.12	4.2
3	2.02	2.53	42.85	14.72	35.78	1.35
4	2.85	5.29	54.95	27.27	2.1	6.24
5	3.01	3.03	44.95	37.21	9.24	1.46
6	3.09	3.85	64.39	17.55	5.02	4.83
7	2.27	3.47	45.53	20.84	23.81	2.44
8	2.64	2.5	5.25	82.35	0.93	5.2
9	12.53	17.46	3.68	47.12	3.84	11.73
10	4.51	6.79	41.23	31.61	3.78	10.49
11	5	4.85	52.67	23.35	6.97	5.66

Analyses revealed several copper-iron-arsenide crystals belonging to the tetragonal and tetragonal dipyramidal systems (1–3, 5, 7), with (8) being precipitating copper globules. Analyses 4, 6, 9, 10, and 11 provide an overview of the region’s general chemistry and are not indicative of any single phase or mineral; however, like all the analyses, they have significant concentrations of Se. Complete EDS datasets are available in S2 Appendix.

## Discussion

Through the application of industrial XCT, this study identified several diagnostic features before the destructive analysis of archaeological slag. Included features were the position and distributions of metal and alloy droplets, pores and voids containing microenvironments, the conditions governing precipitates, and the orientation of the slags as they formed during the smelting process. Regarding the interpretation of the CT scans, in addition to what has already been covered elsewhere in this paper, the magnetite and hematite lining, as well as the pores in slag H76-S39, with densities of 5.11 and 5.14 g/cm³, respectively, sufficiently attenuated the X-rays to mask the inner materials (Fig 4A and 4B).

The presence of these minerals explains why the space within the pore appeared slightly denser than the surrounding air, since, when combined with the predominant fayalite phase in the slag, at 4.47 g/cm^3^, the less dense calcite and gypsum minerals lining the pore, at 2.71 and 2.3 g/cm^3^, respectively, were hidden. In brief, higher-density minerals and phases can obscure or mask lesser ones when uninterrupted high-density mineralizations encircle them [4,9]. This effect, however, can be mitigated by increasing the peak tube voltage [8–10,13,218]. Furthermore, it was shown that for the CuS grains and the intermingled atacamite/paratacamite phases in the droplet in slag H76-S37A, at densities of ~ 4.78 and 3.7 g/cm³, respectively, some indications of different phases and minerals could be seen; however, due to their diminutive size and interspersing, visual separation of them in the CT scan was not possible (Figs 6A, 6B, 12A and 12C). While one cannot identify the intergranular divisions between the covellite grains, discrepancies are evident, shown as non-contiguous internal density differences. In this instance, working inward, the surrounding material is the slag matrix, rich in silicates, followed by the atacamite/paratacamite outer rim and intergranular separations. Internally, CuS and possibly instances of intact Cu_2_S are present, depending on the progression of leaching. For comparison, a small, unaltered, high-density Cu_2_S (~5.65 g/cm³) feature is shown alongside the leached droplet (Fig 6A and 6B), with similar color intensity. In this instance, CT revealed, prior to sectioning, the state of preservation of the leached droplet. For H76-S45B, the picture was far clearer, as the adjacent pores shown around the droplet in the CT scan exactly matched the slag after it was sectioned (Figs 6C, 6D, 15A and 15C). In this instance, the benefits of CT before sectioning are readily apparent. Of note, the internal density of the H76-S45B droplet is more uniform than that of H76-37A, suggesting it was unaltered.

Regarding the location of the droplets relative to the slag orientation, as shown in the CT scans, they were, at least in part, influenced by the cooling rate and viscosity. In general, where cooling occurs rapidly, smaller droplets become entrapped within the silicate glass matrix; conversely, slower cooling, which occurred in this slag, permitted the formation of larger droplets [59,109]. These droplets, influenced by gravity, would then have settled [126], proceeding downward just above the area theorized to have been resting atop a tuyѐre or cylindrical object in slag H76-S45B and the bottom of H76-S37A (*see* S1 Appendix). In these instances, CT non-destructively and non-invasively aided in interpreting the orientation of the slag, while imparting valuable processual and post-depositional information, especially regarding the preservation state and influencing factors that contributed to corrosion, leaching, and secondary precipitates. An obvious example was the discovery of surface-linking channels or cracks in slag H76-S37A, which facilitated the leaching of Cu^2+^ from the copper sulfide droplet and the subsequent formation of atacamite, as illustrated in the Pourbaix diagram.

### Computed tomography limitations

X-ray computed tomography offered several advantages when studying slags; however, caution is necessary when interpreting the results. Relative density in CT refers to the comparison of a material’s density against a reference standard, typically water, which is assigned an HU value of 0 [72], such that an uncorroded metal particle can have a HU value of 4500–10950 while air, at the bottom of the scale, registers as −1000 [10,14,71,73,219]. The relative density of a material dictates the brightness of the corresponding voxels in the scan, and their size, in turn, determines the level of detail and resolution attainable in the reconstructed image [9,72,73,129,218]. Despite fixing the HU values for water and air, voxel intensity cannot currently be used to determine metals, alloy phases, minerals, or necessarily void space, as was encountered in slag H76-S39. A related issue is that of attenuation and the misrepresentation of density, which can be explained by the emission of polychromatic beams and the Beer-Lambert law of exponential attenuation, which defines the relationship between attenuation and the thickness of a homogeneous material where *µ* represents the attenuation coefficient, *I* the intensity of the beam after passing through the object, *I*_*0*_ the initial beam intensity, and *D* the thickness of the object (Equation 14) [220]. The X-rays emitted from the source pass through the sample and are attenuated proportionally to the features and their thicknesses, but, importantly, since only atomic mass and physical density are considered, similar intensities, that is, the energy of the photons, result for different materials [71,72,221]. Attenuated X-rays may also under- or overrepresent density, as they encompass a range of energies. This is due to beam hardening, which causes a proportionally greater number of higher- or lower-energy photons, and by passing through dense materials that hinder the visualization of lower-density materials within [4,9,68,71].


$$\mu =\text{ln}\frac{\left(\frac{I_{\text{0}}}{I}\right)}{D}$$ (14)

High attenuation can hinder the penetration depth of the X-ray beam, making it challenging to image features located deep within objects [9,129]. This limitation is particularly pronounced in dense materials, such as slags and metals, necessitating high-energy X-ray sources for sufficient penetration. Another issue is that while XCT can provide high-resolution images, it may not always capture fine details of artifacts with complex internal structures, such as those with overlapping or superimposed elements. Additionally, the convex surfaces of some artifacts can complicate the scanning process, as the curvature can result in some information being cut off in the image slices, or the streaks and Moiré patterns [6,7,11,71].

### Slag alternation and speiss formation

Returning now to the alteration of the slags, a plethora of secondary precipitates from the smelted anthropogenic phases, as well as the distribution of elements, inhomogeneity, and morphological differences, such as voids and surface-connected conduits, resulted in paragenetic mineralizations in distinct microenvironments that require explanation. As prior scholarship has shown, the slag and glassy silicate matrices are susceptible to weathering [38,40,222]. Sulfides are particularly deleterious, as they generate acidity (Equation 15), which can lead to continued alteration of the slag, exposing more sulfides and dissolving sulfates and metals into solution [40,223,224]. Through weathering, anions such as ${SO}_{\text{4}}^{\text{2}-}$, ${CO}_{\text{3}}^{\text{2}-}$, ${OH}^{-}$, ${PO}_{\text{4}}^{\text{3}-}$, and ${AsO}_{\text{4}}^{\text{3}-}$ and metal cations are released, which in solution favor the precipitation of secondary mineralizations.


$${MeS}_{\text{2}}(s)+\frac{\text{7}}{\text{2}}O_{\text{2}}(aq)+H_{\text{2}}O(l)={Me}^{\text{2}+}(aq)+{\text{2}SO}_{\text{4}}^{\text{2}-}(aq)+{\text{2}H}^{+}(aq)$$ (15)

These secondary minerals are typically oxides and sulfides, but they rarely include iron arsenides such as Fe_2_As, FeAs, and FeAs_2_, which are the speiss phases archaeologists refer to as having formed from pyrometallurgical processes. The first forms in high-temperature melts through a eutectic reaction between the liquid and bcc Fe solid solution at 768–833°C and ~ 24.4 at.% As. The second, from the thermal decomposition of FeAs_2_ between 450–800°C or the eutectoid decomposition of an epsilon phase (~ Fe_3_As_2_) at 824°C and 40 at.% As, and as naturally occurring westerveldite. The last corresponds to the mineral löellingite, and like westerveldite, is stable at ambient temperatures and pressures [58,86,152,153]. Therefore, in a prehistoric copper smelt, Fe_2_As could have formed in the absence of oxygen at high temperatures from a mixture of oxides and sulfides, and remained stable upon cooling [56,58,86], as was likely the case at Shahr-i-Sokhta and Arisman [52,59,209,225–227]. Meanwhile, westerveldite and löellingite, if present, would have likely derived from relics in the ore charge, or they were the result of rapid cooling or quenching, which would have been unlikely given recent reevaluations of copper smelting in Iran [56], meaning that only Fe_2_As can be directly attributed to pyrometallurgy. If quenching had occurred, however, an indicator would be the presence of the NiAs-type polymorph, rather than the MnP-type, of FeAs, which forms at temperatures above 800°C [58]. These facts, along with the previously discussed mechanisms by which As can disseminate within copper smelting slag, raise questions regarding the interpretation of speiss from prehistoric smelting operations. For this research, neither the presence of As and Fe, nor their coexistence in higher concentrations, necessarily indicates that the intended outcome was speiss. Instead, some iron arsenides in the slag are relics, and others likely resulted from mixed ore smelting and processual happenstance. The overall distribution of arsenic in the slag is simply the result of smelting arsenic-rich ores. These ores were further altered through complex corrosion processes, leading to the precipitation of minerals like scorodite from catalysts such as hematite, which were abundant [35,40,213,222,228,229].

## Conclusion

This study incorporated XCT into an archaeological slag study workflow, successfully extracting more information than would have been possible with prescribed analytical approaches and tools alone. Several unique features were discovered, and in-depth interpretations were made possible by interactive 3D images, which enabled the visualization of the overall internal structure of the slags, the distribution of pores, phases, cracks, crevices, and metal droplets, as well as the paragenesis of secondary mineralizations from primary phases and environmental factors in microenvironments. Moreover, while traditional slag analytical methods were employed, the use of XCT proved most fruitful, unmasking regions of interest that were later sectioned, dramatically augmenting not only the pyrometallurgical interpretive outcome but the relationship between the slag’s state of preservation and current chemistry and related mineralizations.

Furthermore, since slags are diagnostic of pyrometallurgical processes and site-specific conditions, it has been shown that comprehensive characterization and interpretation approaches combining metallurgy, geochemistry, corrosion science, and thermodynamics, among other fields, are necessary to draw substantive conclusions. This paper marks the beginning of that endeavor, but more is required to fully elucidate the processes of metal and slag production at Hissar, as well as the relationship between corrosion, leaching, and the post-smelting state of slag artifacts. These slags in particular are the basis of much scholarship in archaeometallurgy, particularly concerning speiss or iron arsenide production, which, here, has only been partially addressed. This work contributes to the corpus of ongoing scholarship on arsenic in prehistoric smelting, reinvigorating the field and offering new, comprehensive perspectives. However, despite the novelty of applied XRT, we must emphasize that traditional analytical techniques are irreplaceable.

This work relied on an array of analytical techniques, which, when combined, confirmed the chemical and mineral phases derived from the source materials, smelting processes, and subsequent interment, leaching, and corrosion activities. Specific ions, such as iron, silicate, and arsenate tetrahedra, along with chlorides and contributions from oxides including arsenic, calcium, and sodium, played distinct roles in elucidating the complexities of the slags and their current state of preservation. Phases formed during smelting, such as magnetite, fayalite, and copper sulfide droplets, along with their high-temperature reactions that sequestered arsenic in calcium arsenates and within the fayalite slag matrix, helped explain prior scholarship on the presence of iron arsenides in ancient Iranian slag, and perhaps more, which is a function of phase stabilities, thermodynamics, and mass transport. The presence of secondary precipitates, such as calcite, atacamite, and scorodite, in the context of thermodynamics and considering environmental conditions in Semnan, Iran, helped determine the types of secondary precipitates identified. Despite the success of XCT in this study, it cannot replace destructive analysis, as it cannot be used to assess phase or composition. Nevertheless, it has now been demonstrated to be indispensable in planning slag analyses and interpreting the significance behind traditional analytical results. In addition to offering a non-destructive and non-invasive method for analyzing the internal structure of slags, the technique further provides digital copies of the artifacts, which are particularly important given their limited number and availability for destructive analyses.

## Supporting information

S1 Appendix Additional slag images. (DOCX)

S2 Appendix Complete EDS chemical datasets. (XLSX)

S3 Appendix R scripts with comments. (MD)

S4 Appendix Thermodynamic data for pourbaix diagrams. (XLSX)

S5 Appendix The distribution of arsenic and formation of iron arsenides. (DOCX)
