# Supplementary figures and images for: A novel application of X-ray computed tomography towards the characterization and interpretation of phase formations, mineral parageneses, and internal features in ancient copper slag from Tepe Hissar, Iran
Source: PLoS One. 2025 Nov 11;20(11):e0336603. doi: 10.1371/journal.pone.0336603 (PMC12604785; doi:10.1371/journal.pone.0336603)

H76-S45B


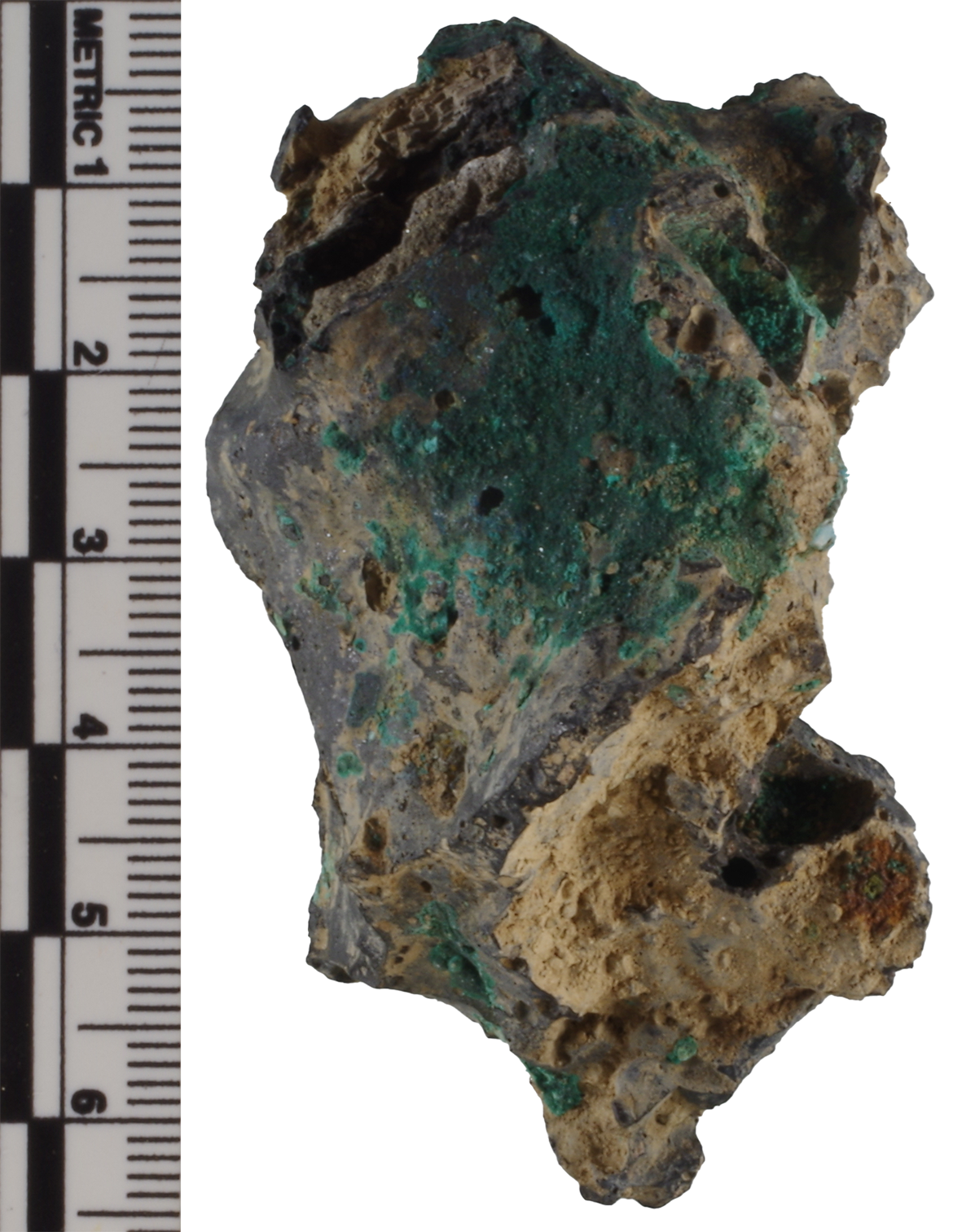


H76-S45B


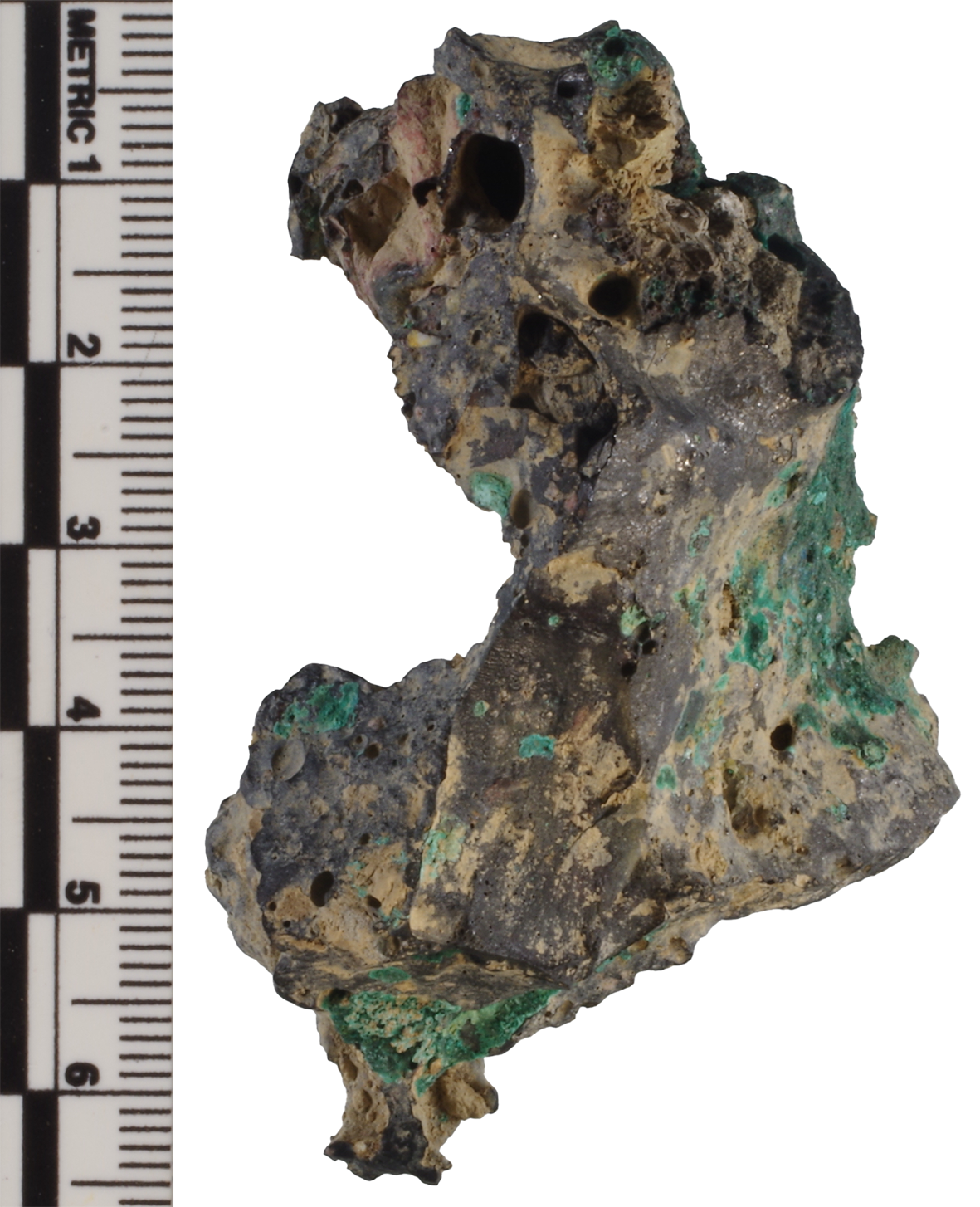


H76-S45B


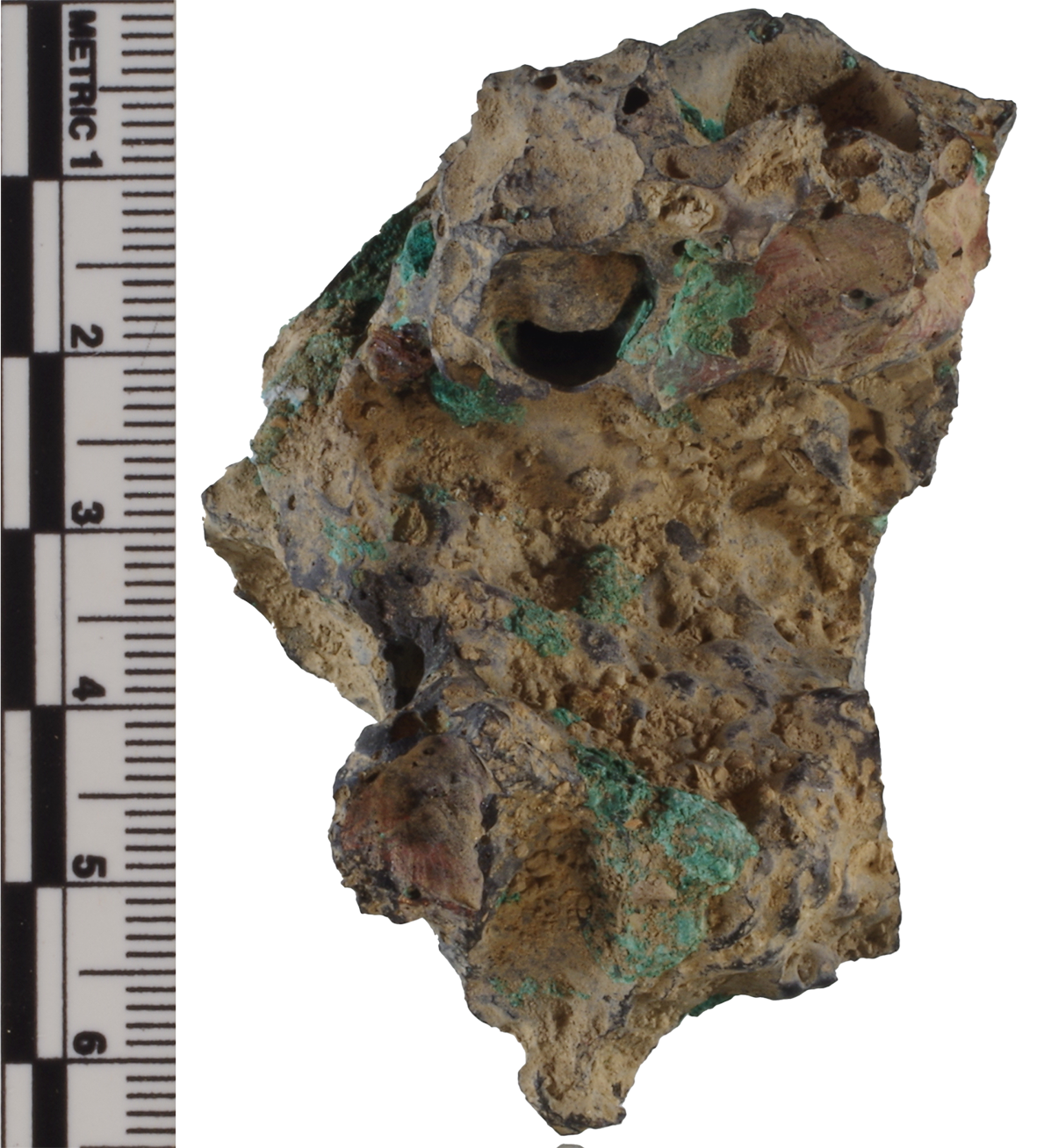


H76-S39


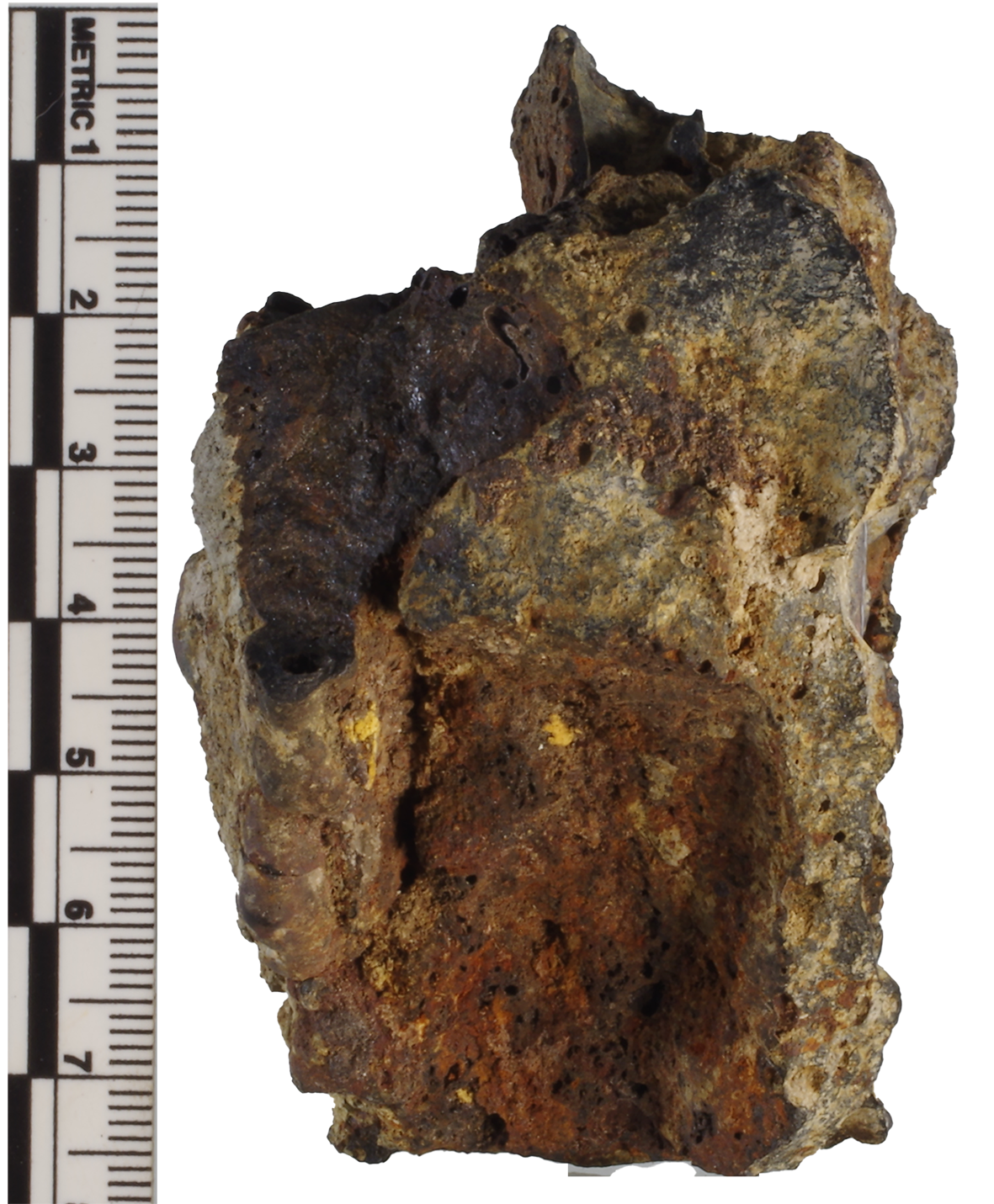


H76-S37A


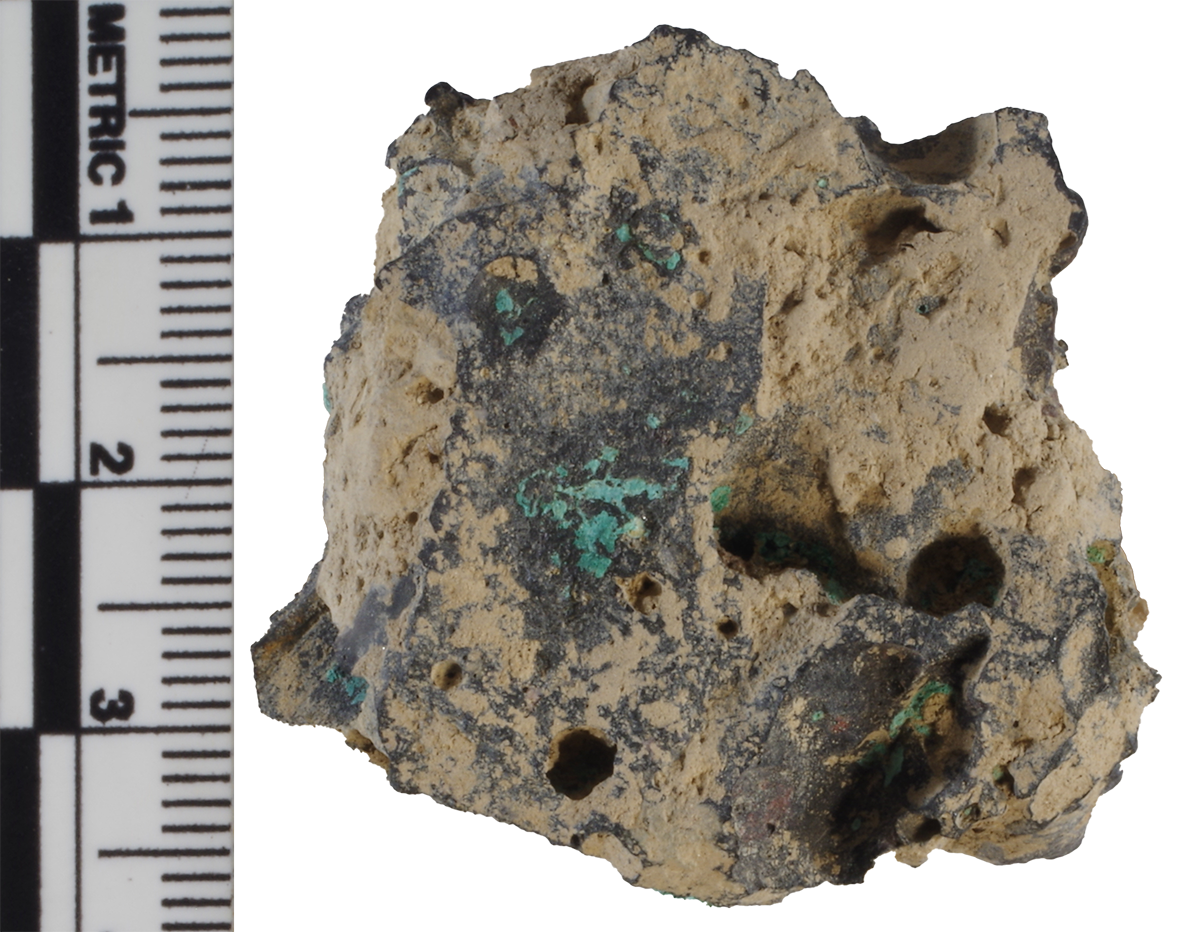


H76-S37A


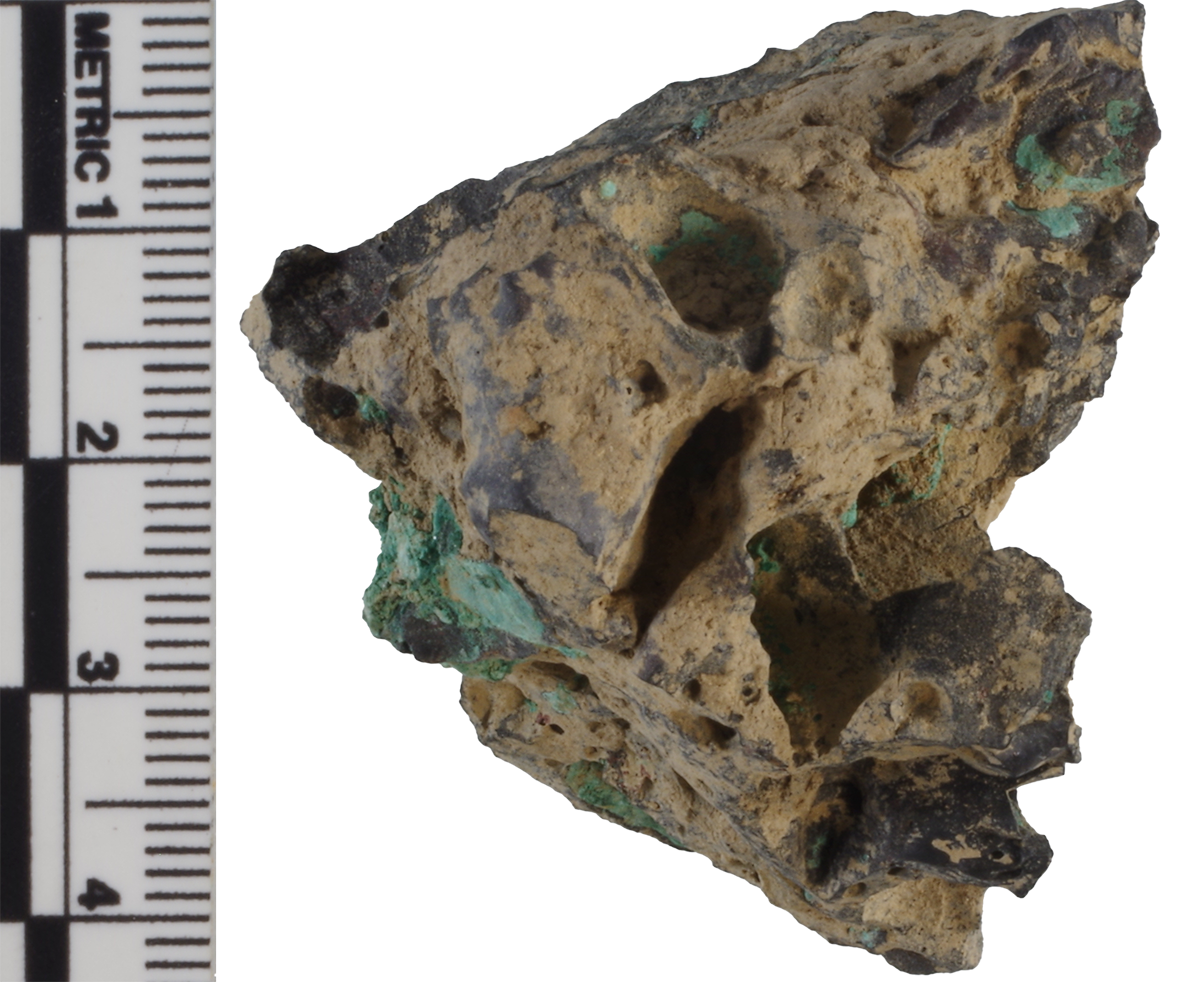


H76-S37A


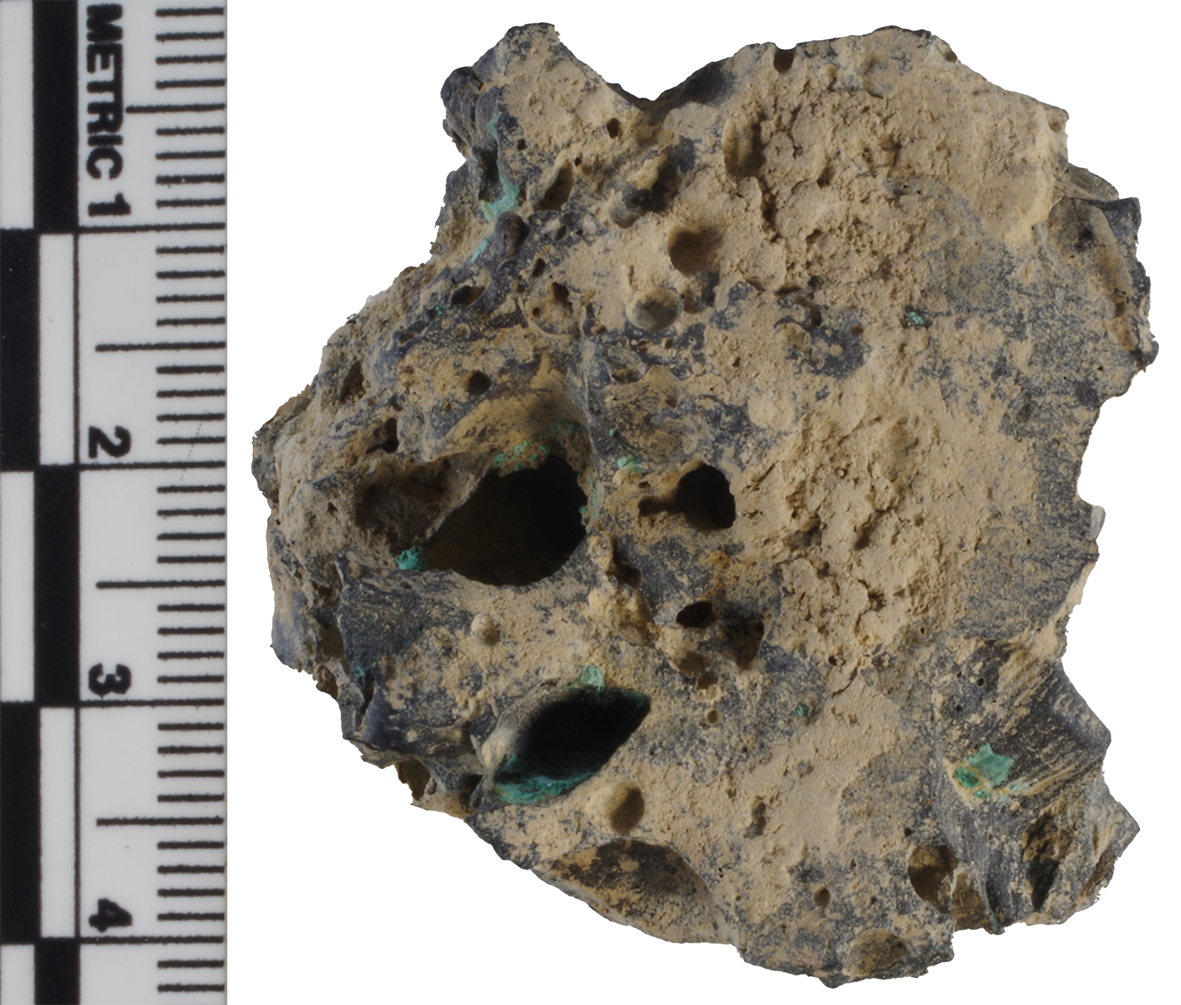


H76-S37A


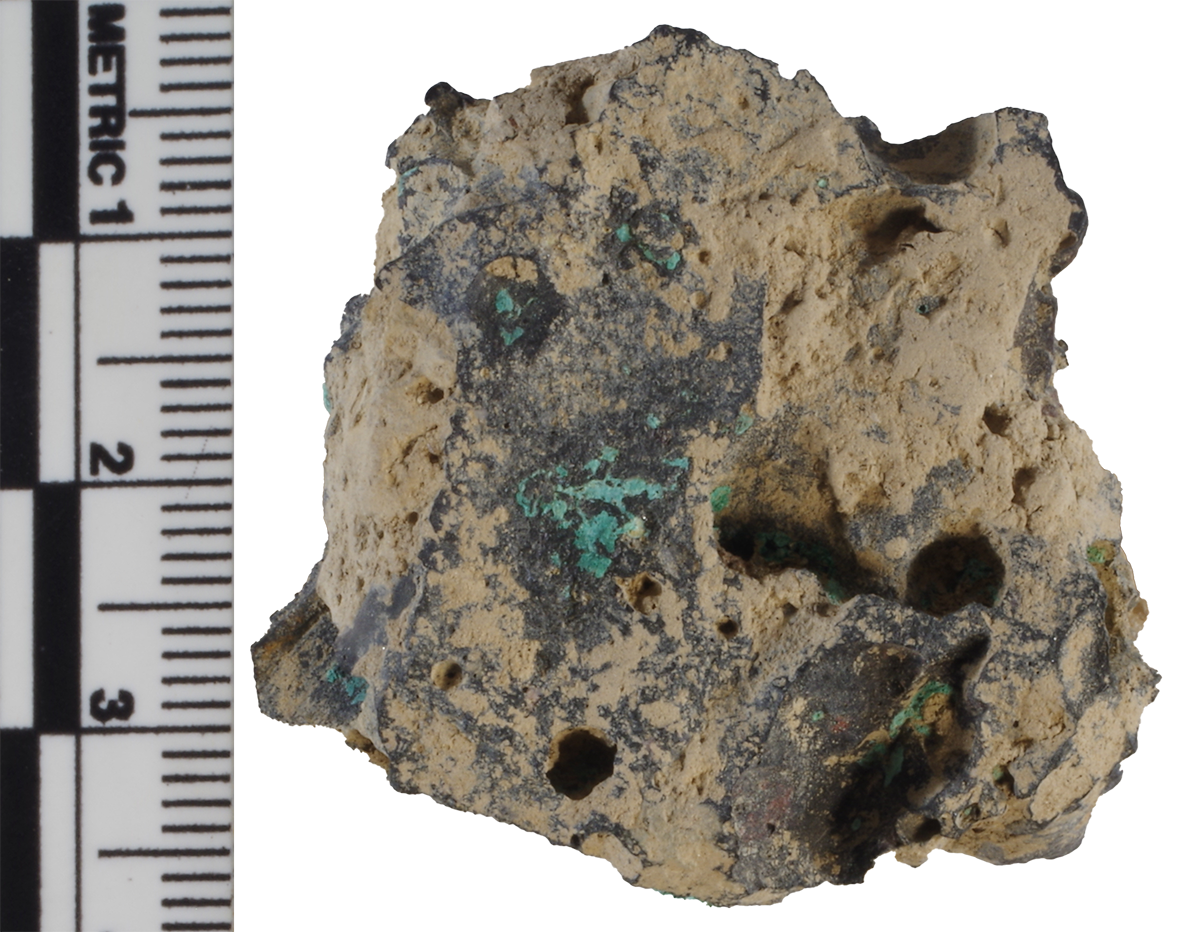

Supplement: S1 Appendix — (DOCX) [file pone.0336603.s001.docx]
