# Supplementary material for: A novel application of X-ray computed tomography towards the characterization and interpretation of phase formations, mineral parageneses, and internal features in ancient copper slag from Tepe Hissar, Iran
Source: PLoS One. 2025 Nov 11;20(11):e0336603. doi: 10.1371/journal.pone.0336603 (PMC12604785; doi:10.1371/journal.pone.0336603)
Supplement: S5 Appendix — (DOCX) [file pone.0336603.s005.docx]

Supplementary S5: The Distribution of Arsenic and Formation of Iron Arsenides

Fluxes, gangue, or associated minerals containing CaO and Na_2_O can influence the redistribution of As during smelting, and thus facilitate the formation of iron arsenides, including Fe_2_As and FeAs, under reducing conditions (1). Sodium (II) would also have been present in solution. The presence of these oxides, whether intentionally added or unknowingly introduced as sulfides in the charge, or by charcoal or pyrotechnic ceramics, alters fayalite phases, eventually leading to the release of As(g), which can react with Fe^2+^ to form FeAs, followed by Fe_2_As (2–4). Calcium (II) or Na^+^ ions act as network modifiers, breaking Si-O-Fe and Fe-O-Fe bonds, releasing FeO and depolymerizing the fayalite structure while generating amorphous sodium- and calcium-silicon in the melt, following Equations 1 and 12 (2,5–12). The FeO can then react with Fe_2_O_3_, already present in the slag, or by partial reduction of Fe (III) to form Fe_3_O_4_ (Equation 3), leading to its enrichment while allowing for Cu metal separation (5). In brief, the amount of magnetite in the slag is related to the amount of free Fe^2+^, which is, in turn, determined by the extent of CaO and Na_2_O-modified fayalite (2).

${Na}_{2}O+{Fe}_{2}{SiO}_{4}\to2\left( FeO \right)+{{Na}_{2}SiO}_{3}$ (1)

$CaO+{Fe}_{2}{SiO}_{4}\to2\left( FeO \right)+{CaSiO}_{3}$ (2)

${Fe}^{2+}+ 2{Fe}^{3+}+{4O}^{2-}\to{Fe}_{3}O_{4}$ (3)

The modifiers furthermore promote the separation of isolated ${SiO}_{4}^{4-}$ tetrahedra, which also contribute to reducing the melt viscosity and liquidus temperature (2,5,13). After the fayalite network is broken, the ${SiO}_{4}^{4-}$ tetrahedra are replaced by ${AsO}_{4}^{3-}$ forming As-O-Si and As-O-Fe bonds across bridging oxygens while depolymerizing the fayalite, leading to a glass-like melt (2,14–16). The ${AsO}_{4}^{3-}$ tetrahedra can form from oxidation, beginning with arsenic sulfide and monomeric arsenic forming As_2_O_3_, followed by arsenic pentoxide (As_2_O_5_), which often coordinates with four oxygens as ${AsO}_{4}^{3-}$ (2,14–16). Concomitantly, the ${AsO}_{4}^{3-}$ tetrahedron can react with FeO from the depolymerized fayalite, and when reduced as Fe^2+^, resulting in As^0^ and Fe^3+^ (Equations 4 and 5); ${FeO}_{4}^{5-}$, like As_2_O_5_, can also coordinate with four oxygen atoms, and by virtue of the Na^+^ and Ca^2+^ modifiers, charge balance is achieved (5,15). In this reaction, the bridging oxygens have a higher binding energy than the non-bridging ones, which contributes to the coprecipitation reaction and the stability of the ${AsO}_{4}^{3-}$ (2).

${Na}_{2}O+2FeO\cdot{SiO}_{2}\left( cr \right)\to2FeO+ {Na}_{2}O\cdot{SiO}_{2} (am)$ (4)

${AsO}_{4}^{3-}+{Fe}^{2+}\to{As}^{0}+ {Fe}^{3+}$ (5)

As the depolymerization reactions show, oxygen has an important role in the availability of As- and Fe-oxides, which can lead to the formation of FeAs and Fe_2_As according to Equations 6 – 9 when smelting polymetallic sulfur-containing ores under highly reducing conditions (1,5,13). Elemental As and Fe can then react to form (di)-iron arsenide; however, as discussed earlier, arsenic is predominantly present in the form of arsenates within the glassy silicate/oxide network of the slag, where it exists as arsenate tetrahedra. And, in the case of sufficiently high CaO, arsenic is more likely to be affixed in the slag as calcium arsenates (2,14,16,17), which were detected by XRD in slag H76-S39 (Fig. 8).

${2Fe}_{2}O_{3}(s)+C(s)\to4FeO(l)+{CO}_{2}(g)$ (6)

$FeO\left( l \right)+C\to Fe+CO(g)$ (7)

$Fe\left( g \right)+As\left( g \right)\to FeAs(s)$ (8)

$FeAs\left( s \right)+CO\left( g \right)+{Fe}_{2}O_{3}(s)\to{Fe}_{2}As(s)+{CO}_{2}(g)$ (9)

References

1. Yang W, Tian S, Wu J, Chai L, Liao Q. Distribution and Behavior of Arsenic During the Reducing-Matting Smelting Process. JOM [Internet]. 2017 Jun 6 [cited 2021 Nov 17];69(6):1077–83. Available from: https://link.springer.com/article/10.1007/s11837-017-2332-8

2. WANG D wei, ZHAO Z wen, LIN Z, LIANG Y jie, KANG L, PENG B. Interaction mechanism between arsenate and fayalite-type copper slag at high temperatures. Transactions of Nonferrous Metals Society of China (English Edition) [Internet]. 2022;32(2):709–20. Available from: http://dx.doi.org/10.1016/S1003-6326(22)65827-8

3. Greener A, Ben-Yosef E. Calcium content in metallurgical slag as a proxy for fuel efficiency of ancient copper smelting technologies. Journal of Lithic Studies [Internet]. 2016;2(1):66–76. Available from: https://www.academia.edu/41410243/Calcium_content_in_metallurgical_slag_as_a_proxy_for_fuel_efficiency_of_ancient_copper_smelting_technologies_UISPPJ_2_1_2019_66_76_

4. Addis A, Angelini I, Nimis P, Artioli G. Late Bronze Age Copper Smelting Slags from Luserna (Trentino, Italy): Interpretation of the Metallurgical Process. Archaeometry [Internet]. 2016 Feb;58(1):96–114. Available from: https://onlinelibrary.wiley.com/doi/10.1111/arcm.12160

5. Liu Y, Xu L, Chen M. Green and efficient recovery of valuable metals from waste copper slag via co-modification with CaO and Na2O. Process Safety and Environmental Protection. 2023;180(October):959–71.

6. Ku J, Zhang L, Fu W, Wang S, Yin W, Chen H. Mechanistic study on calcium ion diffusion into fayalite: A step toward sustainable management of copper slag. J Hazard Mater [Internet]. 2021;410(November 2020):124630. Available from: https://doi.org/10.1016/j.jhazmat.2020.124630

7. Gyurov S, Kostova Y, Klitcheva G, Ilinkina A. Thermal decomposition of pyrometallurgical copper slag by oxidation in synthetic air. Waste Management & Research: The Journal for a Sustainable Circular Economy [Internet]. 2011 Feb 12 [cited 2024 Feb 19];29(2):157–64. Available from: https://pubmed.ncbi.nlm.nih.gov/20705679/

8. Isaksson J, Andersson A, Vikström T, Lennartsson A, Samuelsson C. Improved Settling Mechanisms of an Industrial Copper Smelting Slag by CaO Modification. Journal of Sustainable Metallurgy [Internet]. 2023 Sep 28;9(3):1378–89. Available from: https://link.springer.com/10.1007/s40831-023-00733-x

9. Waseda Yoshio, Toguri JM. The Structure and Properties of Oxide Melts: Application of Basic Science to Metallurgical Processing [Internet]. World Scientific; 1998 [cited 2018 Apr 17]. 236 p. Available from: https://books.google.com/books?id=j626wvLSWSYC&pgis=1

10. Turkdogan ET. Physicochemical Properties of Molten Slags and Glasses [Internet]. Geological Magazine. Metals Society; 1983. 516 p. Available from: https://www.cambridge.org/core/product/identifier/S0016756800031307/type/journal_article

11. Mysen B, Richet P. Silicate glasses and melts. Silicate Glasses and Melts. 2018. 1–720 p.

12. Richardson FD. Physical chemistry of melts in metallurgy. 1974. p. 84.

13. Matinde E, Steenkamp JD. CHAPTER 2. Metallurgical Overview and Production of Slags. In: Metallurgical Overview and Production of Slags [Internet]. The Royal Society of Chemistry; 2021. p. 14–58. Available from: http://dx.doi.org/10.1039/9781788018876-00014

14. Jia W, Li B, Zhou S, Wei Y, Wang H. Distribution behavior and deportation of arsenic in copper top-blown smelting process. Metallurgical Research & Technology [Internet]. 2023 Sep 15 [cited 2024 Feb 19];120(5):507. Available from: https://www.metallurgical-research.org/articles/metal/full_html/2023/05/metal220335/metal220335.html

15. Zhao Z, Wang Z, Xu W, Qin W, Lei J, Dong Z, et al. Arsenic removal from copper slag matrix by high temperature sulfide-reduction-volatilization. J Hazard Mater [Internet]. 2021;415(December 2020):125642. Available from: https://doi.org/10.1016/j.jhazmat.2021.125642

16. Zhang H, Wang Y, He Y, Xu S, Hu B, Cao H, et al. Efficient and safe disposition of arsenic by incorporation in smelting slag through copper flash smelting process. Miner Eng [Internet]. 2021 Jan 1;160:106661. Available from: https://linkinghub.elsevier.com/retrieve/pii/S0892687520304817

17. Riveros G, Utigard TA. Disposal of arsenic in copper discharge slags. J Hazard Mater. 2000;77(1–3):241–52.
